# Detection of Tuberculosis in HIV-Infected and -Uninfected African Adults Using Whole Blood RNA Expression Signatures: A Case-Control Study

**DOI:** 10.1371/journal.pmed.1001538

**Published:** 2013-10-22

**Authors:** Myrsini Kaforou, Victoria J. Wright, Tolu Oni, Neil French, Suzanne T. Anderson, Nonzwakazi Bangani, Claire M. Banwell, Andrew J. Brent, Amelia C. Crampin, Hazel M. Dockrell, Brian Eley, Robert S. Heyderman, Martin L. Hibberd, Florian Kern, Paul R. Langford, Ling Ling, Marc Mendelson, Tom H. Ottenhoff, Femia Zgambo, Robert J. Wilkinson, Lachlan J. Coin, Michael Levin

**Affiliations:** 1 Section of Paediatrics and Wellcome Trust Centre for Clinical Tropical Medicine, Division of Infectious Diseases, Department of Medicine, Imperial College London, London, United Kingdom; 2 Department of Genomics of Common Disease, School of Public Health, Imperial College London, London, United Kingdom; 3 Clinical Infectious Diseases Research Initiative, Institute of Infectious Diseases & Molecular Medicine, University of Cape Town, Cape Town, South Africa; 4 Karonga Prevention Study, Chilumba, Karonga District, Malawi; 5 Institute of Infection & Global Health, University of Liverpool, Liverpool, United Kingdom; 6 Department of Infectious Disease Epidemiology, London School of Hygiene & Tropical Medicine, London, United Kingdom; 7 Brighton and Sussex Medical School, University of Sussex, Brighton, United Kingdom; 8 Malawi-Liverpool-Wellcome Trust Clinical Research Programme, University of Malawi College of Medicine, Blantyre, Malawi; 9 KEMRI-Wellcome Trust Research Programme, Kilifi, Kenya; 10 Department of Immunology and Infection, London School of Hygiene & Tropical Medicine, London, United Kingdom; 11 Red Cross War Memorial Children's Hospital, University of Cape Town, Cape Town, South Africa; 12 Liverpool School of Tropical Medicine, Liverpool, United Kingdom; 13 Infectious Disease, Genome Institute of Singapore, Singapore; 14 Division of Infectious Diseases and HIV Medicine, Department of Medicine, Groote Schuur Hospital, University of Cape Town, Cape Town, South Africa; 15 Department of Infectious Diseases, Leiden University Medical Center, Leiden, The Netherlands; 16 MRC National Institute for Medical Research, London, United Kingdom; 17 Institute for Molecular Bioscience, University of Queensland, St Lucia, Queensland, Australia; San Francisco General Hospital, University of California San Francisco, United States of America

## Abstract

**Background:**

A major impediment to tuberculosis control in Africa is the difficulty in diagnosing active tuberculosis (TB), particularly in the context of HIV infection. We hypothesized that a unique host blood RNA transcriptional signature would distinguish TB from other diseases (OD) in HIV-infected and -uninfected patients, and that this could be the basis of a simple diagnostic test.

**Methods and Findings:**

Adult case-control cohorts were established in South Africa and Malawi of HIV-infected or -uninfected individuals consisting of 584 patients with either TB (confirmed by culture of *Mycobacterium tuberculosis* [M.TB] from sputum or tissue sample in a patient under investigation for TB), OD (i.e., TB was considered in the differential diagnosis but then excluded), or healthy individuals with latent TB infection (LTBI). Individuals were randomized into training (80%) and test (20%) cohorts. Blood transcriptional profiles were assessed and minimal sets of significantly differentially expressed transcripts distinguishing TB from LTBI and OD were identified in the training cohort. A 27 transcript signature distinguished TB from LTBI and a 44 transcript signature distinguished TB from OD. To evaluate our signatures, we used a novel computational method to calculate a disease risk score (DRS) for each patient. The classification based on this score was first evaluated in the test cohort, and then validated in an independent publically available dataset (GSE19491).

In our test cohort, the DRS classified TB from LTBI (sensitivity 95%, 95% CI [87–100]; specificity 90%, 95% CI [80–97]) and TB from OD (sensitivity 93%, 95% CI [83–100]; specificity 88%, 95% CI [74–97]). In the independent validation cohort, TB patients were distinguished both from LTBI individuals (sensitivity 95%, 95% CI [85–100]; specificity 94%, 95% CI [84–100]) and OD patients (sensitivity 100%, 95% CI [100–100]; specificity 96%, 95% CI [93–100]).

Limitations of our study include the use of only culture confirmed TB patients, and the potential that TB may have been misdiagnosed in a small proportion of OD patients despite the extensive clinical investigation used to assign each patient to their diagnostic group.

**Conclusions:**

In our study, blood transcriptional signatures distinguished TB from other conditions prevalent in HIV-infected and -uninfected African adults. Our DRS, based on these signatures, could be developed as a test for TB suitable for use in HIV endemic countries. Further evaluation of the performance of the signatures and DRS in prospective populations of patients with symptoms consistent with TB will be needed to define their clinical value under operational conditions.

*Please see later in the article for the Editors' Summary*

## Abstract 2

Using a microarray-based approach, Michael Levin and colleagues develop a disease risk score to distinguish active from latent tuberculosis, as well as tuberculosis from other diseases, using whole blood samples.

*Please see later in the article for the Editors' Summary*

## Editors' Summary

**Background:**

Tuberculosis (TB), caused by *Mycobacterium tuberculosis*, is curable and preventable, but according to the World Health Organization (WHO), in 2011, 8.7 million people had symptoms of TB (usually a productive cough and fever) and 1.4 million people—95% from low- and middle-income countries—died from this infection. Worldwide, TB is also the leading cause of death in people with HIV. For over a century, diagnosis of TB has relied on clinical and radiological features, sputum microscopy, and tuberculin skin testing but all of these tests have major disadvantages, especially in people who are also infected with HIV (HIV/TB co-infection) in whom results are often atypical or falsely negative. Furthermore, current tests cannot distinguish between inactive (latent) and active TB infection. Therefore, there is a need to identify biomarkers that can differentiate TB from other diseases common to African populations, where the burden of the HIV/TB pandemic is greatest.

**Why Was This Study Done?:**

Previous studies have suggested that TB may be associated with specific transcriptional profiles (identified by microarray analysis) in the blood of the infected patient (host), which might make it possible to differentiate TB from other conditions. However, these studies have not included people co-infected with HIV and have included in the differential diagnosis diseases that are unrepresentative of the range of conditions common to African patients. In this study of patients from Malawi and South Africa, the researchers investigated whether blood RNA expression could distinguish TB from other conditions prevalent in African populations and form the basis of a diagnostic test for TB (through a process using transcription signatures).

**What Did the Researchers Do and Find?:**

The researchers recruited patients with suspected TB attending one clinic in Cape Town, South Africa between 2007 and 2010 and in one hospital in Karonga district, Malawi between 2007 and 2009 (the training and test cohorts). Each patient underwent a series of tests for TB (and had a blood test for HIV) and was diagnosed as having TB if there was microbiological evidence confirming the presence of *Mycobacterium tuberculosis*. At recruitment, each patient also had blood taken for microarray analysis and following this assessment, the researchers selected minimal transcript sets that distinguished TB from latent TB infection and TB from other diseases, even in HIV-infected individuals. In order to help form the basis of a simple, low cost, diagnostic test, the researchers then developed a statistical method for the translation of multiple transcript RNA signatures into a disease risk score, which the researchers then checked using a separate cohort of South African patients (the independent validation cohort).

Using these methods, after screening 437 patients in Malawi and 314 in South Africa, the researchers recruited 273 patients to the Malawi cohort and 311 adults to the South African cohort (the training and test cohorts). Following technical failures, 536 microarray samples were available for analysis. The researchers identified a set of 27 transcripts that could distinguish between TB and latent TB and a set of 44 transcripts that could distinguish TB from other diseases. These multi-transcript signatures were then used to calculate a single value disease risk score for every patient. In the test cohorts, the disease risk score had a high sensitivity (95%) and specificity (90%) for distinguishing TB from latent TB infection (sensitivity is a measure of true positives, correctly identified as such and specificity is a measure of true negatives, correctly identified as such) and for distinguishing TB from other diseases (sensitivity 93% and specificity 88%). In the independent validation cohort, the researchers found that patients with TB could be distinguished from patients with latent TB infection (sensitivity 95% and specificity 94%) and also from patients with other diseases (sensitivity 100% and specificity 96%).

**What Do These Findings Mean?:**

These findings suggest that a distinctive set of RNA transcriptional signatures forming a disease risk score might provide the basis of a diagnostic test that can distinguish active TB from latent TB infection (27 signatures) and also from other diseases (44 signatures), such as pneumonia, that are prevalent in African populations. There is a concern that using transcriptional signatures as a clinical diagnostic tool in resource poor settings might not be feasible because they are complex and costly. The relatively small number of transcripts in the signatures described here may increase the potential for using this approach (transcriptional profiling) as a clinical diagnostic tool using a single blood test. In order to make most use of these findings, there is an urgent need for the academic research community and for industry to develop innovative methods to translate multi-transcript signatures into simple, cheap tests for TB suitable for use in African health facilities.

**Additional Information:**

Please access these websites via the online version of this summary at http://dx.doi.org/ 10.1371/journal.pmed.1001538.

- Wikipedia has definitions of tests for gene expression (note that Wikipedia is a free online encyclopedia that anyone can edit; available in several languages)
- The National Center for Biotechnology Information has a fact sheet on microarray analysis
- MedlinePlus has links to further information about tuberculosis (in English and Spanish)
- The World Health Organization has up-to-date information on TB
- The Stop TB partnership is working towards tuberculosis elimination; patient stories about tuberculosis/HIV coinfection are available

## Introduction

There is an urgent need for improved tests to diagnose active tuberculosis (TB), particularly in countries of sub-Saharan Africa most affected by the TB/HIV pandemic. The diagnosis of TB was problematic even before the emergence of HIV, as symptoms and radiological features of TB overlap those of many other infectious and non-infectious conditions. However in countries of sub-Saharan Africa, where HIV prevalence amongst individuals presenting with symptoms consistent with TB is over 50% [1], the diagnostic difficulty is increased, as TB must be distinguished from a wide range of opportunistic infections and HIV-associated malignancies that present clinically with similar symptoms and signs.

For over a century, diagnosis of TB has relied on clinical and radiological features, sputum microscopy (with or without culture), and tuberculin skin testing (TST). All of these have major drawbacks, particularly in HIV co-infected individuals [2],[3], in whom radiological features are often atypical [4], cavitary lung disease is less common [5],[6], and results of sputum microscopy are often negative [2],[7]. Furthermore, culture facilities are largely unavailable in many African hospitals [8]. As TST and interferon gamma release assays (IGRAs) cannot discriminate TB from latent TB infection (LTBI) [9], they are of limited diagnostic utility amongst African adults where LTBI is highly prevalent in the general population [10], and amongst inpatients with other diagnoses. Molecular methods have improved detection of *Mycobacterium tuberculosis* (M.TB) DNA in sputum [11], but the sensitivity of this approach is lower in smear negative sputum samples even if culture positive [12]. Consequently, high proportions of patients with TB in sub-Saharan Africa remain undiagnosed or are treated empirically without laboratory confirmation. The need for improved diagnostic methods is highlighted by post mortem studies showing TB to be a frequent undiagnosed cause of death in Africa [13]–[15].

RNA expression analysis by microarray has emerged as a powerful tool for understanding disease biology [16]. Many diseases including cancer [17] and infectious diseases [18], as well as TB [19]–[26], are associated with specific transcriptional profiles in blood or tissue. Although previous studies in TB have suggested that RNA expression might be used diagnostically to distinguish TB from other conditions, these studies have excluded HIV-infected participants, and have compared TB with other diseases (OD) that are not representative of the spectrum seen in HIV-infected and -uninfected patients presenting to African hospitals with symptoms for which TB is included in the differential diagnosis [19]–[26]. There is thus a need to identify biomarkers that discriminate TB from OD prevalent in African populations, where the burden of the HIV/TB pandemic is greatest.

In this two country prospective case-control study, we investigated the hypothesis that host peripheral blood RNA expression would distinguish TB from other conditions prevalent in African populations in the context of endemic HIV infection, and explored the use of a transcriptional signature as the basis for a diagnostic test.

## Methods

### Ethics Statement

The study was approved by the Human Research Ethics Committee of the University of Cape Town, South Africa (HREC012/2007), the National Health Sciences Research Committee, Malawi (NHSRC/447), and the Ethics Committee of the London School of Hygiene and Tropical Medicine (5212). Written information was provided by trained local health workers in local languages and all patients provided written consent.

### Study Sites

In order to enable generalization of our findings to African countries with differing prevalence of malaria and other parasitic infections, as well as other environmental exposures that might affect transcriptional profiles, we chose highly contrasting study sites (one urban, one rural) in two African countries with differing co-endemic diseases:

#### Cape Town, South Africa

South Africa has one of the highest TB incidence rates in Africa (981 per 100,000) [27], as well as high rates of HIV infection (up to 41.8% prevalence in females aged 25–35) [28]. Patients undergoing investigation for suspected TB were recruited at GF Jooste Hospital Manenberg, Groote Schuur Hospital, and at Khayelitsha site B clinics serving the largely Xhosa population residing in the low income townships of Cape Town. Malaria is not endemic in these urban populations.

#### Karonga district, Northern Malawi

The incidence of new TB cases in Karonga district (180 per 100,000, Karonga Prevention Study unpublished data, 2012) and the stable HIV prevalence (10%–15% of females aged 25–29, Karonga Prevention Study unpublished data, 2012) are lower in Karonga than in Cape Town. Malaria and helminth infection are hyperendemic. Patients were recruited at Karonga District hospital, which serves a rural population living by the shores of Lake Malawi.

### Diagnostic Process

**Figure 1:**
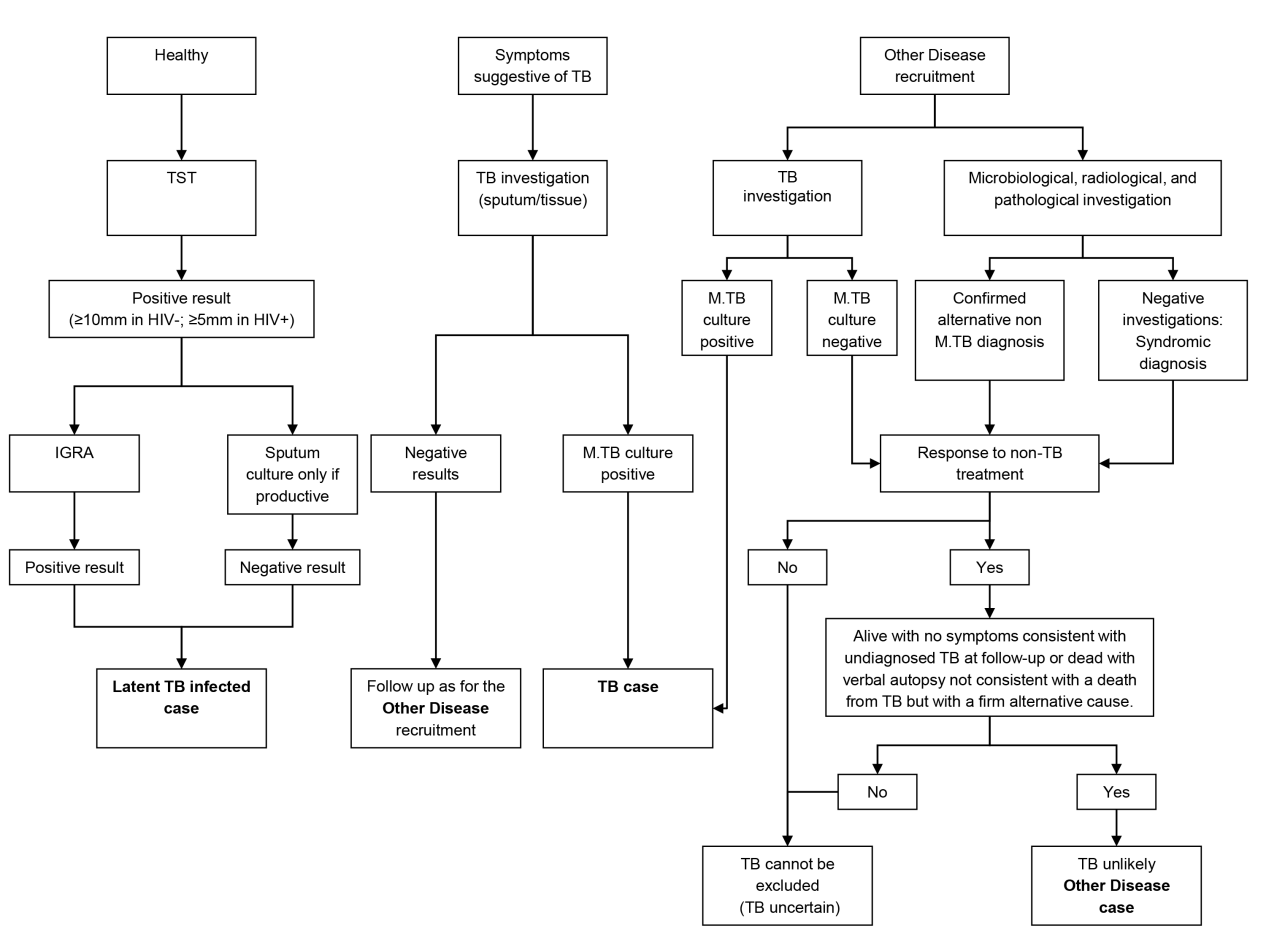
Diagnostic process to identify TB cases, LTBI cases, and other diseases cases.

To ensure accurate assignment of patients to definite TB and OD groups, a rigorous diagnostic process was followed. All patients underwent chest radiographs and serological testing for HIV, along with cultures of blood, CSF, and urine, and biopsies for histological examination including TB culture where clinically indicated. Two sputum samples obtained after induction or coughing were examined by standard microscopy for acid fast bacilli (AFB) and cultured for TB using standard methods (i.e., solid media [South Africa and Malawi] and on liquid media [South Africa only]) [29]. Patients were followed up 26 wk post diagnosis to confirm that those with OD remained TB-free. Individuals were either assigned to one of the diagnostic groups or excluded once the results of investigations and follow-up were available. Healthy LTBI controls were recruited by random community selection (Malawi) and from HIV screening clinics (South Africa) from the same catchment areas as patients with TB (Figure 1). *In vitro* IGRA to substantiate LTBI was undertaken using an in-house whole blood assay [30],[31]. OD patients were recruited if they presented with symptoms that would mandate investigation for TB as a differential diagnosis. After intensive investigation, any case with an established alternative diagnosis to TB, no microbiological evidence of TB, and an absence of TB symptoms at the time of follow-up or with an observed improvement of clinical symptoms on follow-up without TB treatment, was recruited as an OD case. If TB could not be reliably ruled out of the differential, the patient was excluded.

### Patient Cohorts

Patient recruitment strategies, which differed at each site, were embedded within health services administered by statutory providers in order to best investigate on an “intention to test” basis.

#### Cape Town, South Africa

**Figure 2:**
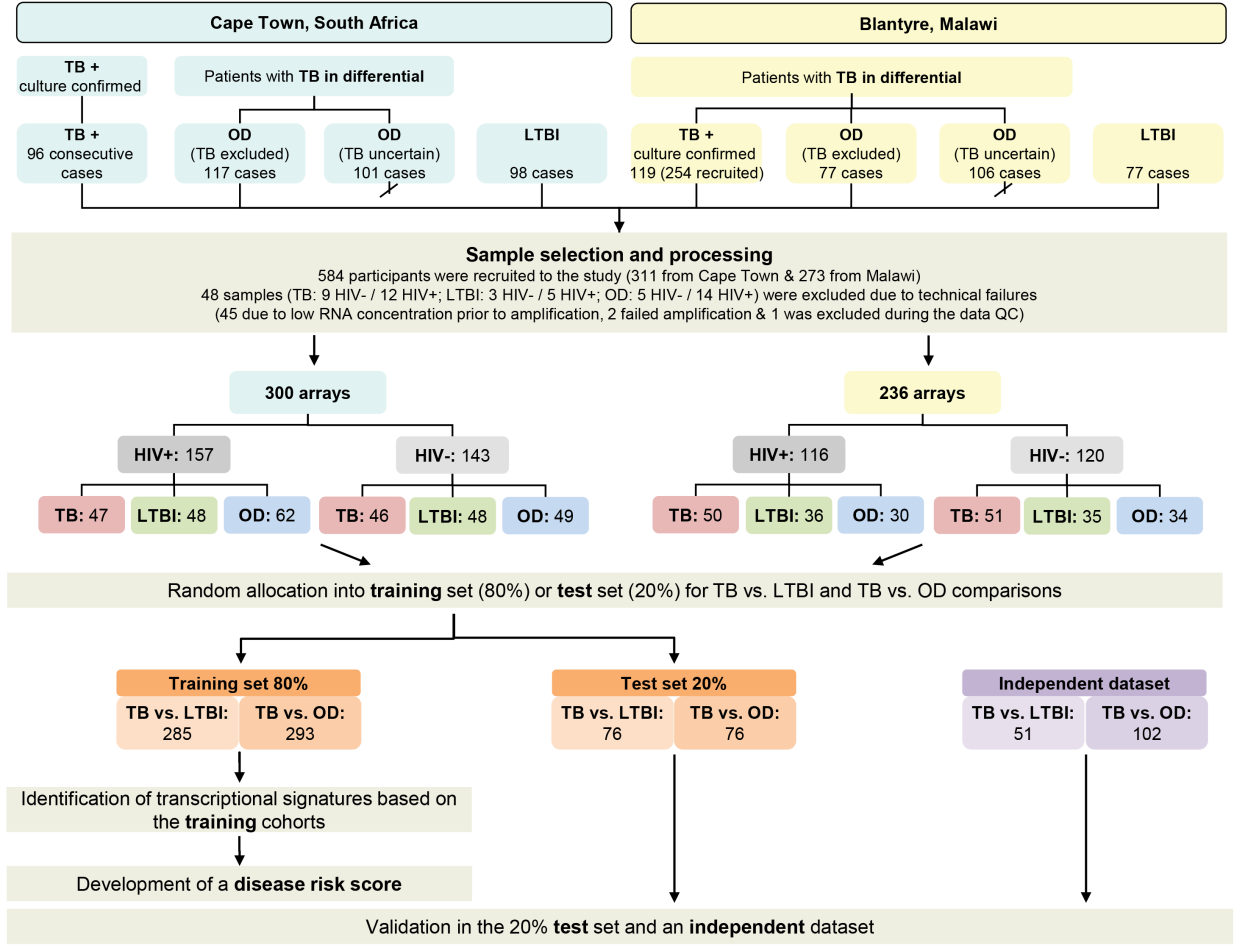
Study overview showing patient numbers and analysis pipeline. HIV-, HIV-uninfected; HIV+, HIV-infected; TB, active tuberculosis (see Table 2).

Recruitment in Cape Town commenced 12th October 2007 and concluded 5th January 2010. Subject to research staff availability, 96 sequential patients presenting with at least one positive TB culture result were recruited from an outpatient TB clinic in Khayelitsha site B until 49 HIV-infected and 47 HIV-uninfected persons were recruited (Figure 2). In Cape Town, 36.7% (18/49) HIV-infected patients with TB were smear-negative and 8.5% (4/47) HIV-uninfected patients were smear-negative. Patients in the OD category were recruited at GF Jooste and Groote Schuur hospitals in Cape Town. Patients were assessed by a hospital clinician and enrolled in the study if TB was considered in the differential diagnosis. After intensive investigation as described above, patients were assigned to the OD group if (1) an alternative diagnosis was established; (2) no microbiological evidence of TB was found after culture of sputum or other samples; and (3) an improvement of clinical symptoms was observed on follow-up without TB treatment (Figure 1). If a patient recruited to the OD group was later found to be culture positive for M.TB, they were reclassified appropriately. In total 138 HIV-infected and 80 HIV-uninfected patients were recruited in the OD group, of which 70 HIV-infected and 31 HIV-uninfected were excluded as TB diagnosis could not be excluded (i.e., TB uncertain) (Figure 2).

#### Karonga district, Northern Malawi

Recruitment at Karonga District Hospital commenced on 1st June 2007 and ceased on the 30th November 2009. Patients attending the hospital were assessed by a local clinician. If this clinician considered TB to be within the differential diagnosis, patients were recruited by a study staff member and investigated according to clinical and study protocols as described above. Following the completion of in-patient care, patients were followed up for at least 26 wk post discharge to assess their progress including a verbal autopsy if the patient had died. Individuals were categorized following the completion of follow-up. Patients were assigned to the OD group if (1) a firm alternative diagnosis was established; (2) there was no microbiological evidence of TB; and (3) there was absence of symptoms of TB at the time of follow-up or assignation of an alternative cause of death on verbal autopsy (Figure 1). Individuals who did not have TB and did not fulfill criteria for OD—e.g., failed to attend follow-up and with an unknown 6-mo outcome—were categorized as “TB uncertain” (i.e., TB uncertain). During the recruitment period 437 patients were recruited. Of these 254 had definite TB, 77 had a confirmed OD, and 106 were categorized as TB cannot be excluded. The first 60 HIV-infected and 59 HIV-uninfected patients with TB, along with all the OD patients were included in the RNA expression study (Figure 2). In Malawi, 13.3% (8/60) HIV-infected patients with TB were smear-negative and 10.2% (6/59) HIV-uninfected patients were smear-negative.

### Oversight and Conduct of the Study

Patients were recruited by FZ and a team of research assistants in Karonga, Malawi, and by TO and hospital staff in Cape Town, South Africa. Assignment of patients to clinical groups was made by consensus of two experienced clinicians at each site (independent of those managing the patient clinically) after review of the investigation results. Testing for HIV status was conducted after appropriate counseling. Clinical data were anonymised and patient samples identified only by study number. Statistical analysis was conducted only after the RNA expression data and the clinical databases had been locked and deposited for independent verification.

### Peripheral Blood RNA Expression by Microarray

Whole blood was collected at the time of recruitment (before or within 24 h of commencing TB treatment in suspected patients) in PAXgene blood RNA tubes (PreAnalytiX), frozen within 3 h of collection, and later extracted using PAXgene blood RNA kits (PreAnalytiX). RNA was shipped frozen to the Genome Institute of Singapore for analysis on HumanHT-12 v.4 expression Beadarrays (Illumina). Additional details of the microarray method, quality control, and analysis are provided in Text S1.

### Statistical Analysis

Expression data were analysed using ‘*R’ Language and Environment for Statistical Computing (R) 2.12.1* (Text S1). To identify transcript signatures applicable across geographic locations and in patients with differing HIV status, we combined HIV-infected and -uninfected patient cohorts from South Africa and Malawi. The recruited participants were randomly assigned to a training cohort (80% of the participants) and a test cohort (20%) with no overlap, using the “sample( )” function without replacement in ‘*R*’, which obtains a subset of a given set [32]. For additional validation we used the whole blood expression dataset from Berry et al. [25] comparing TB with LTBI and other infectious diseases in an African case-control study (accession GSE19491) (i.e., the “validation” dataset) (Text S1).

To detect transcripts that were differentially expressed between patients with TB and comparator groups, a linear model was fitted and moderated t-statistics calculated for each transcript with correction for false discovery using Benjamini and Hochberg's method [33]. Significantly differentially expressed transcripts in the training cohort with a |log_2_ fold change| (FC)>0.5 were subjected to variable selection using elastic net [34] (Text S1) in order to identify the smallest number of transcripts distinguishing TB from the comparator groups. These minimal transcript selected sets for TB versus LTBI and TB versus OD (Tables S1 and S2) were assessed in the test cohort and further evaluated in the validation dataset [25].

### A Simplified Method for Identifying Individual Patient's Risk of Active TB

Current whole genome array-based technologies are not well suited for use in resource poor settings as they are costly and require sophisticated technology as well as bioinformatics expertise. We therefore developed a method for translation of multiple transcript RNA signatures into a disease risk score (DRS), which could form the basis of a simple, low cost, diagnostic test requiring basic laboratory facilities and minimal bioinformatics analysis. For each individual, we calculated (on normalized intensities) the DRS using the minimal transcript selected sets for TB versus LTBI and TB versus OD. The score is derived by adding the total intensity at up-regulated transcripts, and subtracting the total intensity at all down-regulated transcripts (Text S1). The threshold for the classification was calculated as the weighted average of risk score within each class (group of patients), with weights given as the inverse of the standard deviation of the score within each class (Text S1). The information that the DRS requires for classification (i.e., the expression values of the transcripts of the signatures) can be derived from the dataset itself, which allows its unbiased application using expression data acquired using other array platforms or non-array technologies. The sensitivity and specificity of the score in disease classification were evaluated on the test cohort and validation dataset.

### Accession Numbers

The data discussed in this publication have been deposited in NCBI's Gene Expression Omnibus and are accessible through GEO Series accession number GSE37250 (http://www.ncbi.nlm.nih.gov/geo/query/acc.cgi?acc=GSE37250).

## Results

We recruited 311 adults to the South African cohort and 273 to the Malawi cohort meeting the definitions for TB or OD, after screening a total of 314 in South Africa and 437 patients in Malawi (Figures 1 and 2; Table 1). After including samples from LTBI controls that were recruited separately (98 and 77 patients in South Africa and Malawi, respectively) and removing technical failures (48 samples), 536 consecutive patient samples remained for microarray analysis (Figure 2). The spectrum of infectious and malignant diseases in the OD cohorts reflected the range of conditions with similar clinical manifestations to TB at each site (Table 2).

**Table 1:** Clinical and diagnostic features of South Africa and Malawi patients recruited to the study with active tuberculosis, latent TB infection, or other diseases.

Group	TB HIV+		TB HIV−		LTBI HIV+		LTBI HIV−		OD HIV+		OD HIV−	
Location	SA	Malawi	SA	Malawi	SA	Malawi	SA	Malawi	SA	Malawi	SA	Malawi
Number	49	60	47	59	48	41	50	36	68	38	49	39
Age in years median (IQR)	33.7 (29.0–38.3)	34.5 (29.6–43.2)	32.1 (26.3–42.7)	35.6 (26.2–53.1)	31.5 (27.9–37.4)	43.8 (35.4–49.4)	20.6 (19.1–23.4)	38.9 (32.3–50.9)	33.6 (28.6–37.9)	33.8 (29.4–41.3)	40.4 (28.7–53.5)	43.0 (27.0–53.9)
Sex (male, %)	40	52	70	58	27	22	42	53	38	34	45	28
Duration of symptoms/days median (IQR)	21 (0–33)	60 (14–210)	30 (21–30)	60 (30–240)	NA	NA	NA	NA	21 (6–90)	7 (3–90)	42 (7–130)	7 (2–365)
BMI (kg/m^2^) median (IQR)	22.6 (19.5–25.2)	18.5 (16.9–20.7)	19.5 (18.0–22.5)	18.7 (16.5–20.2)	24.2 (20.6–28.4)	21.2 (18.6–23.9)	22.2 (21.4–25.7)	22.0 (20.2–23.4)	21.4 (20.0–24.6)	19.8 (18.3–22.2)	22.6 (18.4–24.9)	21.1 (19.6–22.2)
CD4 count/mm^3^ median (IQR)	174 (64.7–293)^a^	128 (35–314)	NA	NA	326 (231–555)	312 (240–418)	NA	NA	197 (92–357)^b^	198 (111–270)	NA	NA
Anti-retroviral therapy	4 (8%)	14 (23.3%)	NA	NA	1 (2%)	0 (0%)	NA	NA	26 (38.2%)	16 (42.1%)	NA	NA
Tuberculin skin test induration (mm) median (IQR)	20 (15.5–22)^c^	ND	ND	ND	16 (10–20)	17 (0–25)	15 (12–20)	13 (11–17)	ND	0 (0–0)	ND	0 (0–9)
IGRA positive (see Methods)	ND	ND	ND	ND	48 (100%)	22 (53.7%)	50 (100%)	13 (36.1%)	ND	ND	ND	ND
Malaria positive	NA	2 (3.3%)	NA	2 (3.4%)	NA	1 (2.4%)	NA	0 (0%)	NA	3 (7.9%)	NA	2 (5.1%)

BMI, body mass index; HIV−, HIV-uninfected; HIV+, HIV-infected; IQR, inter quartile range; LTBI, latent TB infection; NA, not applicable; ND, not done; OD, other diseases (see Table 2); SA, South Africa; TB, active TB;

a Four missing values.

b Ten missing values.

c 33 missing values, not routinely performed in the work up of TB+/HIV+ patients.

**Table 2:** Major clinical diagnoses in other diseases cohorts.

Other Diseases	HIV Infected		HIV Uninfected		Total
	SA	Malawi	SA	Malawi	
Pneumonia/LRTI/PJP	24 (35%)	19 (50%)	5 (10%)	13 (33%)	61 (31%)
Malignancy and other neoplasia other than Kaposi's sarcoma^a^	2 (3%)	4 (11%)	17 (35%)	5 (13%)	28 (14%)
Pelvic inflammatory disease/UTI	4 (6%)	1 (3%)	15 (31%)	5 (13%)	25 (13%)
Bacterial, viral meningitis, or meningitis of uncertain origin	4 (6%)	4 (11%)	0 (0%)	6 (15%)	14 (7%)
Hepatobiliary disease	6 (9%)	0 (0%)	7 (14%)	0 (0%)	13 (7%)
Febrile syndromes of uncertain origin	1 (1%)	3 (8%)	1 (2%)	6 (15%)	11 (6)%
Kaposi's sarcoma	9 (13%)	1 (3%)	0 (0%)	0 (0%)	10 (5%)
Cryptococcal meningitis	6 (9%)	4 (11%)	0 (0%)	0 (0%)	10 (5%)
Non TB pleural effusion/empyema	5 (7%)	0 (0%)	2 (4%)	0 (0%)	7 (4%)
Gastroenteritis	5 (7%)	0 (0%)	0 (0%)	0 (0%)	5 (3%)
Peritonitis	0 (0%)	1 (3%)	0 (0%)	3 (8%)	4 (2%)
Other^b^	0 (0%)	1 (3)%	2 (4%)	1 (3%)	4 (2%)
Gastric ulcer or gastritis	2 (3%)	0 (0%)	0 (0%)	0 (0%)	2 (1%)
Total	**68**	**38**	**49**	**39**	**194**

a Bronchial carcinoma (14), lymphoma (4), cervical carcinoma (1), ovarian carcinoma (1), mesothelioma (1), gastric carcinoma (1), metastatic carcinoma of unknown origin (4), benign salivary tumour (1), dermatological tumour (1).

b HIV-related lymphadenopathy(1), Crohn′s disease (1), orchitis (1), pyomyositis (1).

LRTI, lower respiratory tract infection; PJP, *Pneumocystis jirovecii* pneumonia; SA, South Africa; UTI, urinary tract infection.

### TB Specific RNA Signature That Is Independent of Geographic Location and HIV Status

We performed quality control on the microarray data in order to examine the effect of disease state on transcript expression and to check for assignment errors. Inspection revealed that the primary clustering was based on disease state (TB, LTBI, OD) rather than geographical location or HIV status (Figure S1). There was substantial correlation of TB versus LTBI differential expression across different geographic locations and HIV status, which was also seen for TB versus OD (Figures S2 and S3). This indicates the presence of a robust underlying signature of TB, independent of HIV status or geographical location.

### Identification and Validation of Minimal Transcript Sets

**Figure 3:**
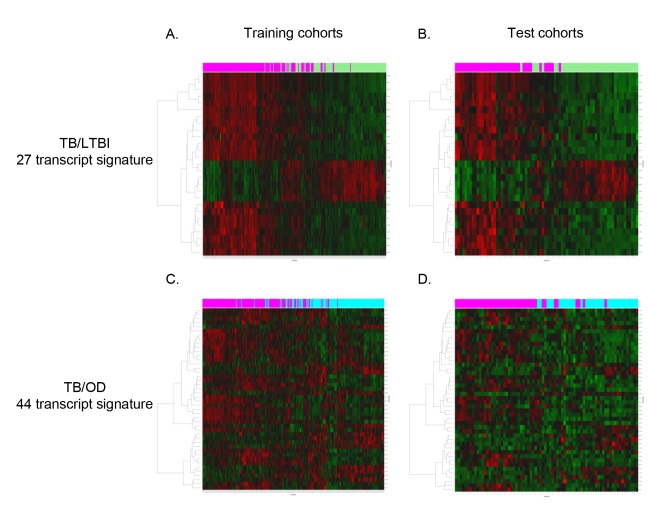
Heatmaps showing clustering of training and test cohorts using transcriptional signatures. Clustering of training (A/C) and test (B/D) cohorts using transcripts identified by elastic net for TB versus LTBI (A/B) and TB versus OD (C/D) (training: *n*_TB_ = 157 *n*_LTBI_ = 128/*n*_TB_ = 153 *n*_OD_ = 140, test: *n*_TB_ = 37 *n*_LTBI_ = 39/*n*_TB_ = 42 *n*_OD_ = 34). Rows are transcripts (transcripts shown in red are up-regulated, those in green are down-regulated) and columns are patients regardless of HIV status (purple, patients with TB; green, patients with LTBI; light blue, patients with OD).

To find minimal transcript sets required to discriminate TB from other groups, we applied the variable selection algorithm elastic net [34] to the training cohort (Methods; Text S1). A 27 transcript model was identified for discriminating TB from LTBI in the South Africa/Malawi training and test set (Figure 3A and 3B; Table S1), whilst a 44 transcript model was identified for discriminating TB from OD (Figure 3C and 3D; Table S2). These models were also applied to data from the South Africa validation dataset [25], which, unlike our cohort, included only HIV-uninfected participants (Figure S4).

### Evaluation of a Simplified Disease Risk Score for TB

**Figure 4:**
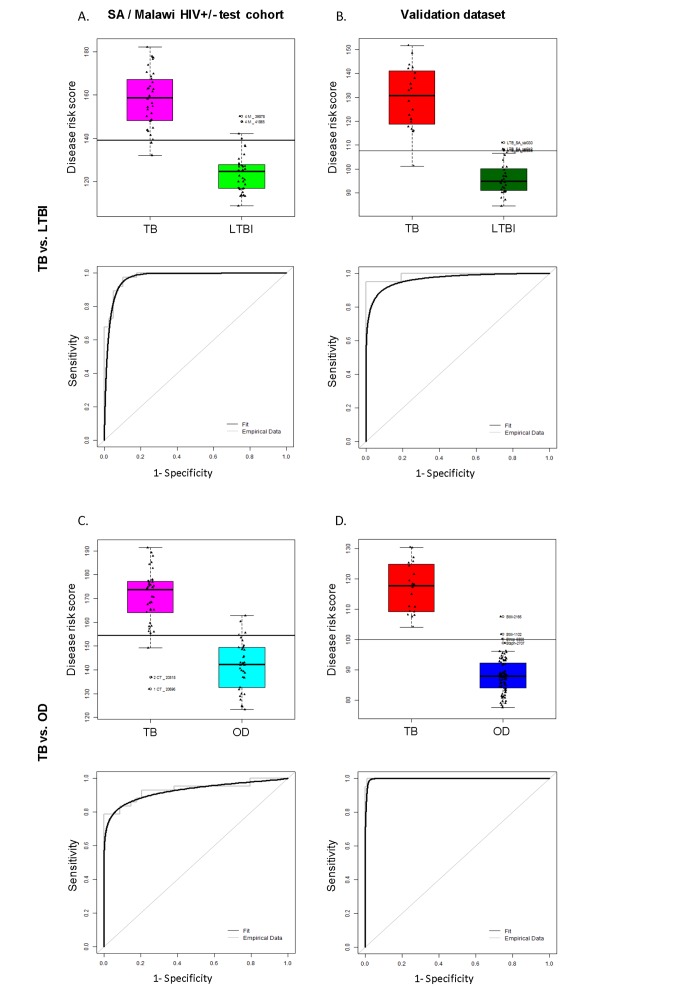
Classification using the disease risk score on the test cohort and validation dataset. Disease risk score and receiver operating characteristic curves based on the TB/LTBI 27 transcript signature (A/B) and the TB/OD 44 transcript signature (C/D) applied to the South African (SA)/Malawi HIV+/− test cohort (A/C) (*n*_TB_ = 37 *n*_LTBI_ = 39/*n*_TB_ = 42 *n*_OD_ = 34) and independent validation dataset comprising South African patients (B/D) (*n*_TB_ = 20 *n*_LTBI_ = 31 *n*_OD_ = 82). Sensitivity, specificity are reported in Table 3. HIV+, HIV-infected; HIV−, HIV-uninfected. Classification cut-offs: (A) 138.98; (B) 107.76; (C) 154.44; (D) 99.94.

**Figure 5:**
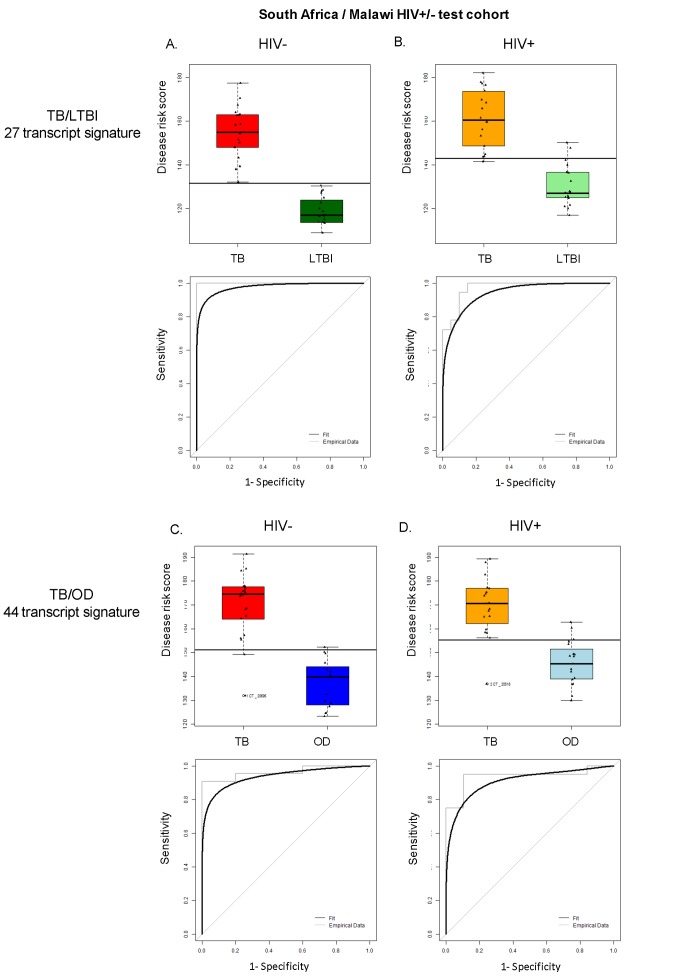
Application of the transcript signatures to the South African and Malawi test cohorts by HIV status. Disease risk score and receiver operating characteristic curves based on the TB/LTBI 27 transcript signature (A/B) and the TB/OD 44 transcript signature (C/D) applied to the HIV-uninfected (HIV−) (A/C) and HIV-infected (HIV+) (B/D) test cohort. Area under the curve, sensitivities, and specificities are reported in Table 3. Classification cut-offs: (A) 131.37; (B) 142.84; (C) 151.10; (D) 142.84.

To evaluate the feasibility of using a simplified diagnostic test based on our transcript sets for TB diagnosis in low resource settings, we applied the DRS to our test cohort, which includes patients that were not used to discover the signatures, and to the South Africa validation dataset [25]. In our combined HIV-infected and -uninfected test set, the 27 transcript DRS discriminated TB from LTBI with sensitivity and specificity of 95%, 95% CI (87–100), and 90%, 95% CI (80–97), respectively, whilst achieving perfect classification in the HIV-uninfected cohorts and a slightly reduced accuracy in the HIV-infected cohorts (Figures 4A, 5A, and 5B; Table 3). In the validation dataset, the DRS achieved a sensitivity of 95%, 95% CI (85–100), and a specificity of 94%, 95% CI (84–100) (Figure 4B; Table 3). As for the discrimination between TB and OD, the 44 transcript DRS's sensitivity and specificity were 93%, 95% CI (83–100), and 88%, 95% CI (74–97), respectively, with consistent accuracy in the HIV-infected and -uninfected test cohorts (Figures 4C, 5C, and 5D; Table 3). In the validation dataset, the patients were classified with 100% sensitivity, 95% CI (100–100), and 96% specificity, 95% CI (93–100) (Figure 4D; Table 3). Similar values for sensitivity and specificity were obtained when the DRS was evaluated in the training dataset, demonstrating the robustness of our approach to avoid overfitting (Table S5). In order to evaluate the classificatory power of the DRS, we compared its performance with the regression model derived from the elastic net based on the same signatures (Table S5). We found that our DRS had similar accuracy in distinguishing TB from LTBI and OD to the weighted regression model.

**Table 3:** Classification achieved using the disease risk score.

Measures	South Africa/Malawi Test Cohort			Validation Dataset
	HIV+/− (95% CI)	HIV− (95% CI)	HIV+ (95% CI)	HIV− (95% CI)
**TB versus LTBI (27 TB/LTBI transcript signature)**				
Number of patients	76	38	38	51
Area under the curve	98% (95–100)	100% (100–100)	97% (95–100)	99% (97–100)
Sensitivity	95% (87–100)	100% (100–100)	94% (83–100)	95% (85–100)
Specificity	90% (80–97)	100% (100–100)	90% (75–100)	94% (84–100)
Likelihood ratio positive	9.23 (3.63–23.4)	NA	9.44 (2.52–5.34)	14.73 (3.84–56.47)
Likelihood ratio negative	0.06 (0.02–0.23)	0	0.06 (0.01–0.42)	0.05 (0.01–0.36)
**TB versus ODs (44 TB/OD transcript signature)**				
Number of patients	76	37	39	102
Area under the curve	95% (89–99)	96% (89–100)	94% (83–100)	100%^a^ (100–100)
Sensitivity	93% (83–100)	91% (77–100)	95% (85–100)	100% (100–100)
Specificity	88% (74–97)	93% (80–100)	84% (68–100)	96% (93–100)
Likelihood ratio positive	7.89 (3.13–19.89)	14.3 (2.15–95.12)	6.02 (2.1–17.08)	27.67 (9.11–84.03)
Likelihood ratio negative	0.08 (0.03–0.24)	0.05 (0.01–0.35)	0.06 (0.01–0.41)	0

The TB/LTBI 27 transcript signature and TB/OD 44 transcript signature were applied to the South African/Malawi HIV-uninfected (HIV−) and HIV-infected (HIV+) test cohort and the independent validation dataset. Sensitivity and specificity calculated using the weighted threshold for classification. The actual numbers of patients that were DRS negative and positive are shown in Table S4.

^a^99.94%.

HIV−; HIV-uninfected; HIV+; HIV-infected; NA; not applicable.

In order to assess the predictive value of our DRS in a cohort of patients undergoing investigation for persistent symptoms such as cough, fever, and weight loss, i.e., where TB was included in the differential diagnosis, we used the prevalence of TB in our prospective Malawi cohort (58%; 254 confirmed TB cases of 437 patients with suspected TB) to calculate the positive and negative predictive value (PPV/NPV). The DRS for TB versus OD had a PPV of 92%, 95% CI (84–99), and a NPV of 90%, 95% CI (80–100) (Table S7). Using a 20% prevalence, which may be more reflective of a general primary care setting in a high-burden African country, NPV for TB versus OD is higher (98%, 95% CI [96–100]), but PPV decreases (66%, 95% CI [46–87]), emphasizing the value of DRS as a rule-out test, with those patients with positive DRS selected for further investigation (Table S7).

We also explored the effect of adjusting the threshold for the DRS in assigning individual patients to TB or LTBI/OD. By accepting a percentage of patients as “non-classifiable,” the majority of patients under investigation are accurately assigned. These “non-classifiable” patients could then be selected for more detailed investigation (Figure S5).

As it would be advantageous to have a single signature that distinguished TB from non-TB, we assessed the performance of a signature in distinguishing TB from both TB and LTBI. A 53 transcript signature was identified (Table S3) that distinguished TB from both LTBI and OD with sensitivity/specificity 91%/82%—a lower performance than TB/LTBI and TB/OD signatures alone. We also explored whether a smaller number of transcripts could be used to distinguish TB from LTBI and from OD, which would aid in manufacturing of a test (Text S1), resulting in a 21 and 29 transcript signature for distinguishing TB from LTBI and OD, respectively. The sensitivity of the smaller models was 6%–10% lower than the original models, while retaining the same specificity for TB versus OD (Table S8).

**Figure 6:**
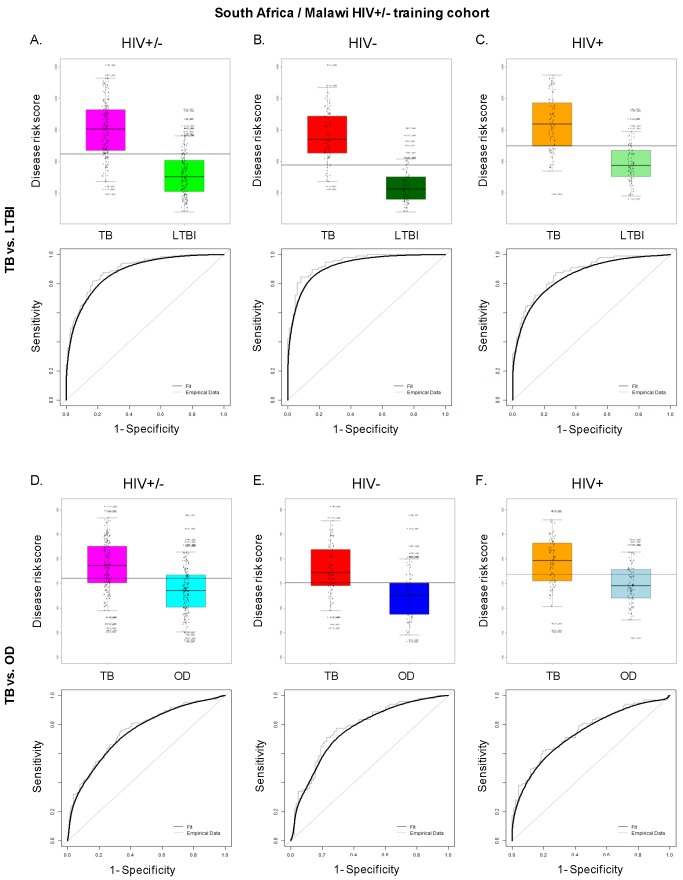
Application of transcript signatures [**25**] to the combined South Africa and Malawi cohorts. Disease risk score and receiver operating characteristic curves based on transcript signatures of Berry et al. [25] for TB versus LTBI (A/B/C) and TB versus OD (D/E/F) applied to the combined training and test cohorts in HIV-uninfected (HIV−) and HIV-infected (HIV+) (A/D), HIV− (B/E), and HIV+ (C/F) cohorts (Table 4 for sensitivities, specificities, and area under the curve). Classification cut-offs: (A) 1,847.73; (B) 1,777.65; (C) 1,898.97; (D) 172.12; (E) 170.30; (F) 173.70.

In contrast to our approach, previous studies of RNA expression as a diagnostic tool for TB have excluded HIV-infected patients, and have used other disease controls that were not recruited concurrently with TB cases or from the same population of patients undergoing investigation for TB [19],[21],[22],[24],[25]. To establish how these differences in biomarker study design might affect performance of biomarker signatures, we compared the performance of our 27 transcript TB/LTBI signature and our 44 transcript TB/OD signature with the performance of the signatures of Berry et al. [25] for discrimination of TB versus LTBI (393 transcripts) and TB versus OD (86 transcripts). While the 393 TB/LTBI signature achieved a sensitivity of 88%, 95% CI (80–94), and a specificity of 84%, 95% CI (76–92), on our TB HIV-uninfected cohorts, the performance on the HIV-infected group was 74%, 95% CI (65–82), and 80%, 95% CI (71–87), respectively (Figure 6; Table 4). Furthermore, the Berry et al. TB/OD 86 transcript signature had a lower performance on our cohorts (sensitivity 71%, 95% CI (62–80), specificity 76%, 95% CI (67–84), in HIV-uninfected; sensitivity 67%, 95% CI (58–75), specificity 69%, 95% CI (59–78), in HIV-infected) (Figure 6; Table 4). Thus our minimal transcript signatures and the DRS method show better performance in distinguishing TB from LTBI and OD (especially in the HIV-infected cohorts) than the much larger number of transcripts identified by Berry et al. [25]. (Table 5)

**Table 4:** Application of published signatures to the South Africa and Malawi cohorts.

Measures	South African/Malawi Cohorts		
	HIV−/+ (95% CI)	HIV− (95% CI)	HIV+ (95% CI)
**TB versus LTBI (393 transcript signature)**			
Number of patients	361	180	181
Area under the curve	89% (86–92)	94% (91–97)	88% (82–92)
Sensitivity	82% (76–87)	88% (80–94)	74% (65–82)
Specificity	81% (75–87)	84% (76–92)	80% (71–87)
**TB versus OD (86 transcript signature)**			
Number of patients	369	180	189
Area under the curve	76% (70–80)	78% (70–84)	75% (68–82)
Sensitivity	68% (61–73)	71% (62–80)	67% (58–75)
Specificity	70% (62–76)	76% (67–84)	69% (59–78)

Sensitivities, specificities, and area under curve based on transcript signatures of Berry et al. [25] for TB versus LTBI (393 transcripts), and TB versus OD (86 transcripts) applied to the South African/Malawi HIV-uninfected (HIV−) and HIV-infected (HIV+) cohorts.

**Table 5:** Performance of the TB/LTBI 27 and TB/OD 44 transcript signatures and the transcript signatures of Berry et al. [25] when applied to our test cohort.

Measures	South Africa/Malawi Test Cohort								
	HIV+/− (95% CI)			HIV− (95% CI)			HIV+ (95% CI)		
	Our Signatures	Berry et al. Signatures	Difference^a^	Our Signatures	Berry et al. Signatures	Difference^a^	Our Signatures	Berry et al. Signatures	Difference^a^
**TB versus LTBI**									
**Area under the curve**	98%	88%	+10%	100%	91%	+9%	97%	89%	+9%
	(95–100)	(85–97)	(2–18)	(100–100)	(88–100)	(0–18)	(92–100)	(83–98)	(−3 to 20)
**Sensitivity**	95%	84%	+11%	100%	90%	+11%	94%	78%	+17%
	(87–100)	(73–95)	(1–21)	(100–100)	(74–100)	(1–20)	(83–100)	(61–94)	(2–32)
**Specificity**	90%	87%	+3%	100%	79%	+21%	90%	85%	+5%
	(80–97)	(77–97)	(−8 to 13)	(100–100)	(58–95)	(8–34)	(75–100)	(65–100)	(−10 to 20)
**TB versus OD**									
**Area under the curve**	95%	73%	+22%	96%	76%	+20%	94%	72%	+21%
	(89–99)	(63–86)	(10–33)	(89–100)	(62–91)	(5–35)	(82–100)	(57–89)	(5–37)
**Sensitivity**	93%	74%	+19%	91%	77%	+14%	95%	70%	+25%
	(83–100)	(60–86)	(8–31)	(77–100)	(59–96)	(−3 to 30)	(85–100)	(50–90)	(9–41)
**Specificity**	88%	74%	+15%	93%	67%	+27%	84%	74%	+11%
	(74–97)	(59–88)	(2–27)	(80–100)	(40–87)	(9–44)	(68–100)	(53–90)	(−7 to 28)

Comparison of the statistical measures of performance of disease classification using our TB/LTBI 27 and TB/OD 44 transcript signatures with the classification using the 393 (−6 transcript) and 86 (−1 transcript) transcript signatures from Berry et al. [25]. The marked improvement shown for HIV+ individuals in both TB versus LTBI and TB versus OD comparisons suggests that transcript signatures must be derived from both HIV-infected and -uninfected individuals in order to have a diagnostic value in these populations. The performance of our signatures in TB versus OD comparison highlights the need for real world “other disease” controls when deriving biomarkers from clinical cohorts.

a Calculations of the differences were performed before rounding for reporting purposes on the paper.

Finally, we evaluated the performance of our signatures in the smear-negative sub-group of patients with TB, the majority of whom were HIV-infected (31 smear-negative TB patients with definite negative smear status; seven TB HIV-uninfected and 24 TB HIV-infected). In the smear-negative patients the DRS showed a sensitivity for detecting TB of 68%, 95% CI (52–84), when using the TB versus LTBI signature and a sensitivity of 90%, 95% CI (81–100), with the TB/OD signature, both of which are comparable to results obtained in the larger HIV-infected cohort of smear-positive and -negative patients. As we used the same LTBI and OD patients from the test set, the specificity was unchanged (90%, 95% CI (80–97), for TB versus LTBI and 88%, 95% CI (74–97), for TB versus OD) (Table S9).

## Discussion

We have identified a host blood transcriptomic signature that distinguishes TB from a wide range of OD prevalent in HIV-infected and -uninfected African patients. We found that patients with TB can be distinguished from LTBI with only 27 transcripts and from OD with 44 transcripts. Our findings appear robust as the results are reproducible in both HIV-infected and -uninfected cohorts, in different geographic locations, and in an independent TB patient dataset. The high sensitivity and specificity of the signatures in distinguishing TB from OD, even in the HIV-infected patients that have differing levels of T cell depletion and a wide spectrum of opportunistic infections as well as HIV-related complications, suggests that the signatures are promising biomarkers of TB. The relatively small number of transcripts in our signatures may increase the potential for using transcriptional profiling as a clinical diagnostic tool from a single peripheral blood sample (i.e., using a multiplex assay [35],[36]).

The major challenge for diagnosis of TB in Africa is how to distinguish this disease from the range of other conditions that show similar symptoms in countries where TB and HIV are co-endemic. Previous TB biomarker studies have focused on distinguishing patients with TB from healthy controls, or from LTBI [21],[22],[24], or have used other disease controls that may not represent the “real world” disease spectra from which TB should be clinically differentiated [19],[25]. Furthermore, these TB biomarker studies have also excluded HIV co-infected patients who are the group that most need new diagnostics. Our study design should ensure that our signatures are applicable in TB/HIV endemic countries as we recruited patients with TB concurrently with patients with a range of conditions that present with similar clinical features to TB, as well as recruiting both HIV-infected and -uninfected individuals.

We have identified separate signatures for distinguishing TB/OD and TB/LTBI, which only overlap in three transcripts. In practice the clinical applications of these signatures might be distinct as the TB/LTBI signature would be of value in contact screening, where the concern is distinguishing active disease from previous exposure in minimally symptomatic individuals. The TB/OD signature would be of most value in evaluating symptomatic patients presenting to medical services with symptoms of TB. We have also explored whether a single signature might be used to distinguish TB from both LTBI and OD. The combined signature showed lower performance to the separate TB/LTBI and TB/OD signatures. Further exploration of the operational performance of a combined signature or separate signatures is needed to establish the best strategy.

Although our signatures and DRS distinguished the majority of patients with TB from those with LTBI or OD, a proportion of patients were not correctly classified. There is increasing recognition that TB and LTBI may represent a dynamically evolving continuum, particularly in HIV-infected patients and thus failure to culture M.TB is not absolute proof that TB is not present. Some false assignment by our current “gold standard” is to be expected as noted by post mortem studies at which undiagnosed TB is confirmed [14],[15]. All patients in the OD group presented with symptoms for which TB was included in the differential diagnosis, and it is possible that TB may have been misdiagnosed in a small proportion of OD patients despite the extensive clinical investigation used to assign each patient to each diagnostic group. Some improvement in sensitivity and specificity of our DRS may also be achieved by weighting the signal from the most discriminatory transcripts, and this could be explored in subsequent refinements of the method.

A major concern in using transcriptional signatures as a clinical diagnostic tool in resource poor settings is the complexity, as well as cost, of the current methodologies. Our results have shown that transcriptional signatures can be used to distinguish TB from OD in an African setting. We explored the feasibility of a simplified method for disease categorization that may facilitate development of a diagnostic test based on our signatures. Our DRS provides a new approach that enables the use of multi-transcript signatures for individual disease risk assignment without the requirement for complex analysis. Our method could be used to develop a simple test in which the transcripts comprising the diagnostic signature (separated into those that are either up- or down-regulated in TB relative to controls) are each measured using a suitable detection system [35], and the combined signature used to identify each patient's risk of TB. For example, a simple test using the TB/OD signature probes that show increased transcript expression in TB relative to OD could be located in a single well or tube, and those probes that show reduced transcript expression in TB located in a second well or tube. Binding of RNA from a patient's blood to these probes could be detected as a combined signal from each tube using one of the aforementioned detection systems. To allow normalization, expression of up- or down-regulated transcripts in an individual patient could be compared with that of housekeeping genes, which do not show variation between healthy and disease states. There are methods for rapid detection of multi-transcript signatures including lateral flow reverse transcription (RT)-PCR based systems, nano-pore technology [37], nano-particle enzyme linked detection [38],[39], and detection using nano-wires and electrical impedance [40]. Some of these may be suitable for direct analysis of multiple transcript signatures in blood and at a relatively low cost.

While this study provides a proof of principle that relatively small numbers of RNA transcripts can be used to discriminate active TB from latent TB infection and OD in Africa, limitations remain that need to be addressed in order to translate these results into a clinical test. One such limitation is that our study has not assessed performance of our DRS in patients treated for TB solely on the basis of clinical suspicion, without any microbiological confirmation. Amongst these “probable/possible” patients with TB, there is no gold standard to evaluate any new biomarker. Exclusion of probable/possible patients with TB may have produced better estimates of sensitivity and specificity than would be achieved in a prospective “all comers” study including the entire cohort of patients in whom TB is included in the differential diagnosis. Thus, further evaluation using a prospective population based study in which the decision whether and when to initiate TB treatment is evaluated against the new biomarker is required. Future studies will also be required to refine the use of these biomarkers in a clinical decision process either as an initial screening tool, or in conjunction with more detailed culture based diagnostics.

From a clinical perspective a simple transcriptome-based test that reliably diagnoses or excludes TB in the majority of patients undergoing investigation for suspected TB, using a single blood sample, would be of great value, allowing scarce hospital resources to be focused on the small proportion of patients where the result was indeterminate. The challenge for the academic research community and for industry is to develop innovative methods to translate multi-transcript signatures into simple, cheap tests for TB suitable for use in African health facilities.

## Supporting Information

Figure S1 **Principal components analysis (PCA) of the microarray samples.** PCA plot based on all transcripts on all samples after background adjustment and normalisation. A) PCA1 & PCA2 and B) PCA1 & PCA3. The sample highlighted (categorised as active TB HIV+ from Malawi) was removed from the analysis. Rings are levels of confidence (0.9 inner circle, 0.9999 outer circle).
(TIF)

Figure S2 **Concordance of differential expression by location of cohort and by HIV status for TB versus LTBI.** Concordance of differential expression by location of cohort (A/B) and by HIV status (C/D) for the active TB versus latent TB infection cohorts in South Africa and Malawi. Negative logarithm of the corrected p-values in TB versus LTBI between South Africa and Malawi for HIV-uninfected (HIV−) cohort (A) and HIV-infected (HIV+) cohort (B); and between HIV− and HIV+ cohorts in South Africa (C) and in Malawi (D). There were positive correlations between all comparisons. p = 0.05 is equivalent to −log p value = 1.3.
(TIF)

Figure S3 **Concordance of differential expression by location of cohort and by HIV status for TB versus OD.** Concordance of differential expression by location of cohort (A/B) and by HIV status (C/D) for the active TB versus other disease cohorts in South Africa and Malawi. Negative logarithm of the corrected p-values in TB versus OD between South Africa and Malawi for HIV-uninfected (HIV−) cohort (A) and HIV-infected (HIV+) cohort (B); and between HIV− and HIV+ cohorts in South Africa (C) and in Malawi (D). There were positive correlations between all comparisons. Note, the correlation between South Africa/Malawi HIV− cohorts is less than in South Africa/Malawi HIV+ cohorts which may reflect the different spectra of conditions in the ‘other disease’ cohorts. p = 0.05 is equivalent to −log p value = 1.3.
(TIF)

Figure S4 **Heatmaps showing clustering of the independent South African validation dataset based on the TB/LTBI and TB/OD signatures.** Clustering of TB versus LTBI based on the TB/LTBI 27 transcript signature (A) and TB/OD 44 transcript signature (B) applied to the independent South African validation datasets of Berry et al. [25]. Patients are represented as columns (red are patients with TB, green are LTBI, blue are OD) and individual transcripts are shown in rows (transcripts shown in red are up-regulated and those in green are down-regulated).
(TIF)

Figure S5 **Calculating the error rate of the classifiers.** The error rate of classification is presented in relation to the percentage of unclassified samples. We present the error rate of the classifier for the different groups using the 27 TB/LTBI and 44 TB/OD transcript signatures in relation to the missing rate we accept (HIV+ patients in red, HIV− in blue and both HIV+ & HIV− in black; solid lines show the error rate for the training cohorts while dotted lines show the error rate for the test cohorts).
(TIF)

Table S1 **The 27 transcript signature for distinguishing TB from LTBI.**
(DOC)

Table S2 **The 44 transcript signature for distinguishing TB from other diseases.**
(DOC)

Table S3 **The 53 transcript signature for detecting TB from non-TB (i.e., LTBI and OD).**
(DOC)

Table S4 **Number of patients per group and calls of DRS classification per group.**
(DOC)

Table S5 **Comparison of classification achieved using elastic net derived linear classifier and disease risk score for every pairwise comparison.**
(DOC)

Table S6 **Classification achieved using the disease risk score applied to the South African/Malawi HIV-uninfected (HIV−) and HIV-infected (HIV+) test cohort and validation dataset with confidence intervals calculated using the exact binomial method (Text S1).**
(DOC)

Table S7 **Positive and negative predictive values for the classification achieved using the disease risk score applied to the South African/Malawi HIV-uninfected (HIV−) and HIV-infected (HIV+) test cohort and validation dataset.**
(DOC)

Table S8 **Performance of the smaller signatures when applied to the South Africa/Malawi test set.**
(DOC)

Table S9 **Classification achieved using the disease risk score applied to the South African/Malawi smear-negative patients with TB and the controls from the test cohort with confidence intervals calculated using the bootstrapping and the exact binomial method.**
(DOC)

Text S1 **Appendix.**
(DOC)

Text S2 **STARD checklist.**
(DOC)

## Abbreviations

DRS: disease risk score
IGRA: interferon gamma release assay
LTBI: latent tuberculosis infection
OD: other diseases
TB: tuberculosis
TST: tuberculin skin testing
