# Supplementary figures and images for: Detection of Tuberculosis in HIV-Infected and -Uninfected African Adults Using Whole Blood RNA Expression Signatures: A Case-Control Study
Source: PLoS Med. 2013 Oct 22;10(10):e1001538. doi: 10.1371/journal.pmed.1001538 (PMC3805485; doi:10.1371/journal.pmed.1001538)

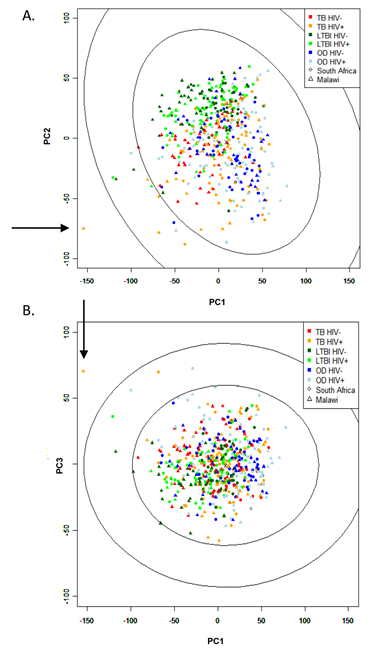

Supplement: Figure S1 — Principal components analysis (PCA) of the microarray samples. PCA plot based on all transcripts on all samples after background adjustment and normalisation. A) PCA1 & PCA2 and B) PCA1 & PCA3. The sample highlighted (categorised as active TB HIV+ from Malawi) was removed from the analysis. Rings are levels of confidence (0.9 inner circle, 0.9999 outer circle). (TIF) [file pmed.1001538.s001.tif]

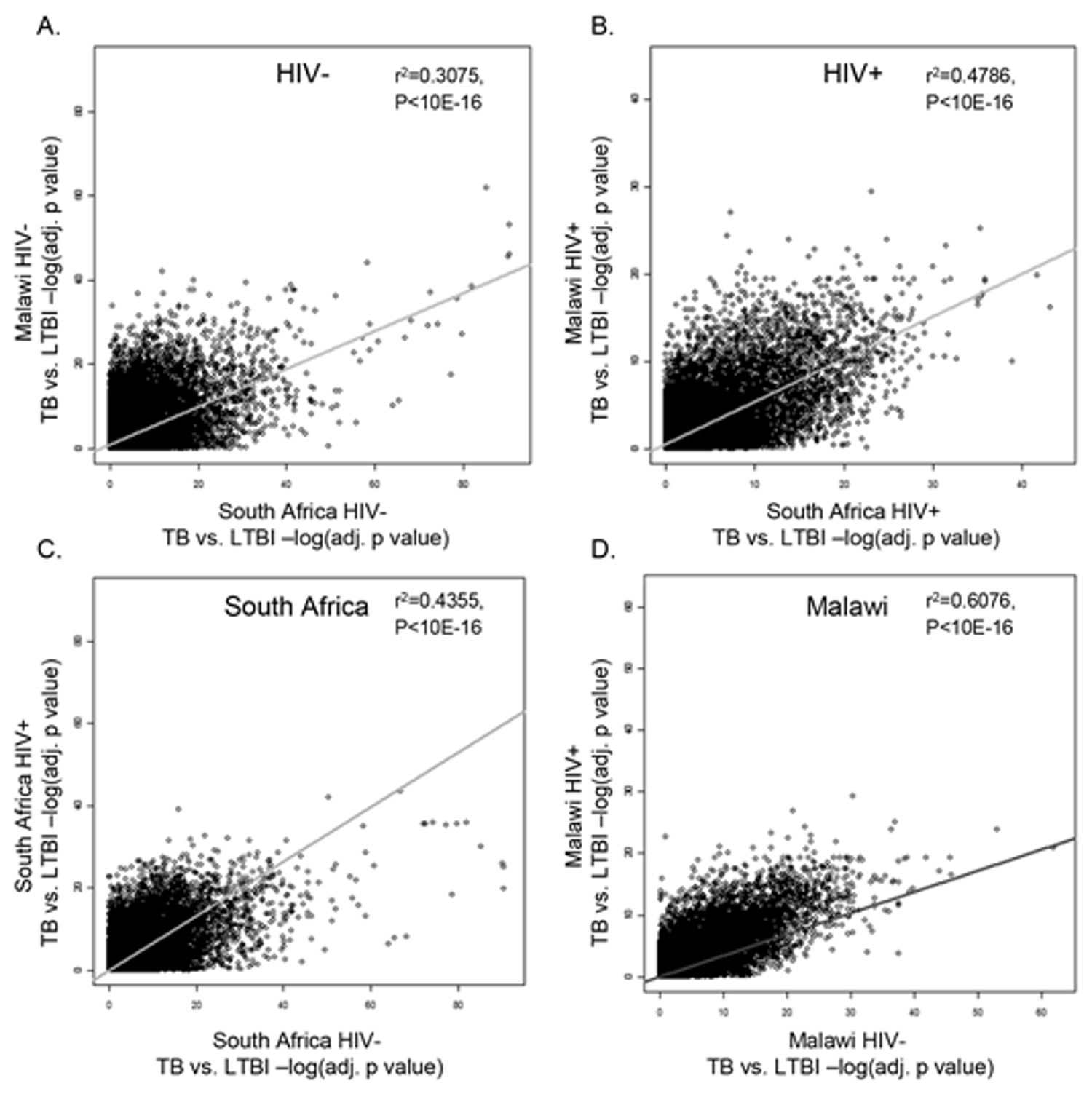

Supplement: Figure S2 — Concordance of differential expression by location of cohort and by HIV status for TB versus LTBI. Concordance of differential expression by location of cohort (A/B) and by HIV status (C/D) for the active TB versus latent TB infection cohorts in South Africa and Malawi. Negative logarithm of the corrected p-values in TB versus LTBI between South Africa and Malawi for HIV-uninfected (HIV−) cohort (A) and HIV-infected (HIV+) cohort (B); and between HIV− and HIV+ cohorts in South Africa (C) and in Malawi (D). There were positive correlations between all comparisons. p = 0.05 is equivalent to −log p value = 1.3. (TIF) [file pmed.1001538.s002.tif]

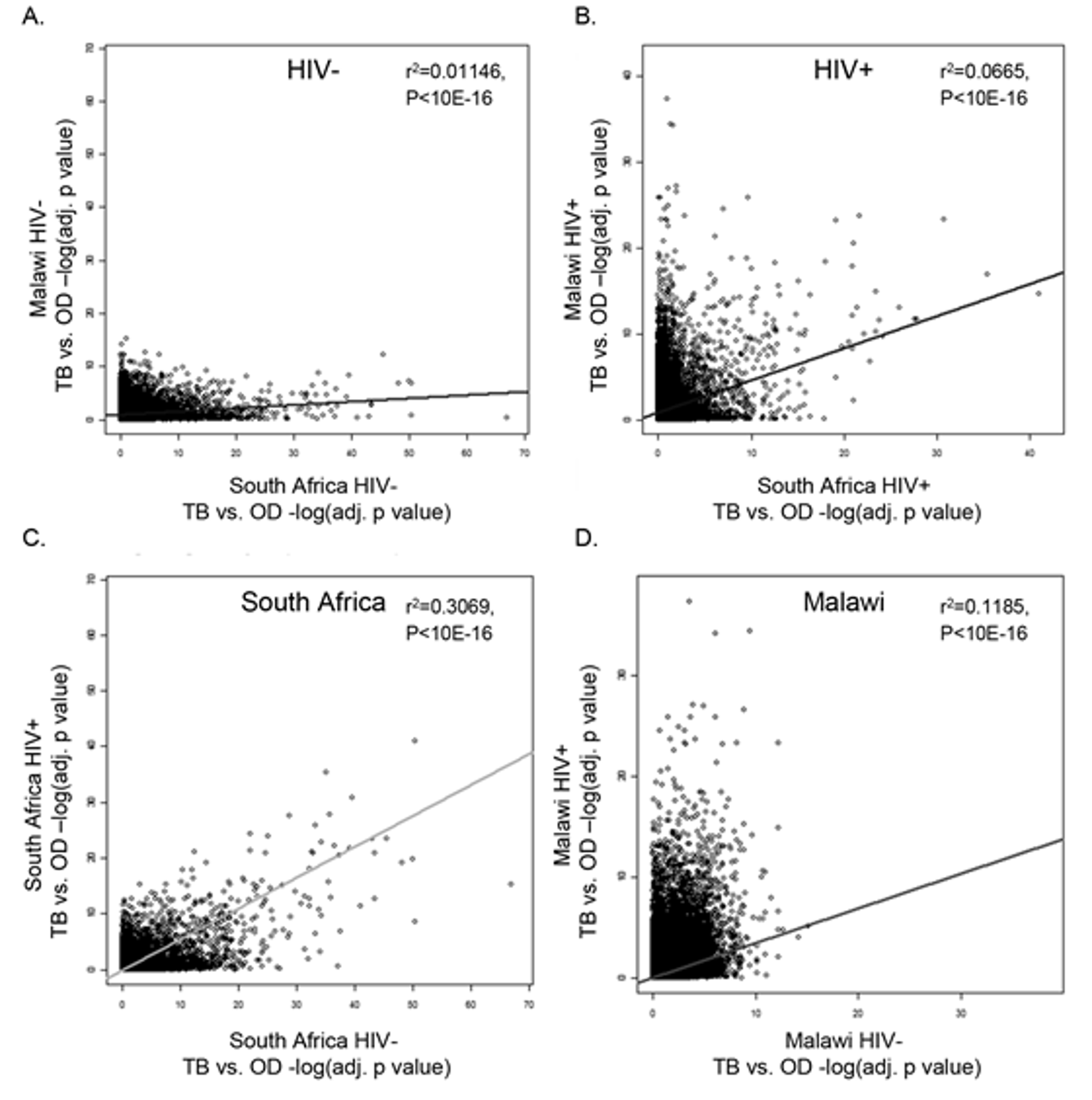

Supplement: Figure S3 — Concordance of differential expression by location of cohort and by HIV status for TB versus OD. Concordance of differential expression by location of cohort (A/B) and by HIV status (C/D) for the active TB versus other disease cohorts in South Africa and Malawi. Negative logarithm of the corrected p-values in TB versus OD between South Africa and Malawi for HIV-uninfected (HIV−) cohort (A) and HIV-infected (HIV+) cohort (B); and between HIV− and HIV+ cohorts in South Africa (C) and in Malawi (D). There were positive correlations between all comparisons. Note, the correlation between South Africa/Malawi HIV− cohorts is less than in South Africa/Malawi HIV+ cohorts which may reflect the different spectra of conditions in the ‘other disease’ cohorts. p = 0.05 is equivalent to −log p value = 1.3. (TIF) [file pmed.1001538.s003.tif]

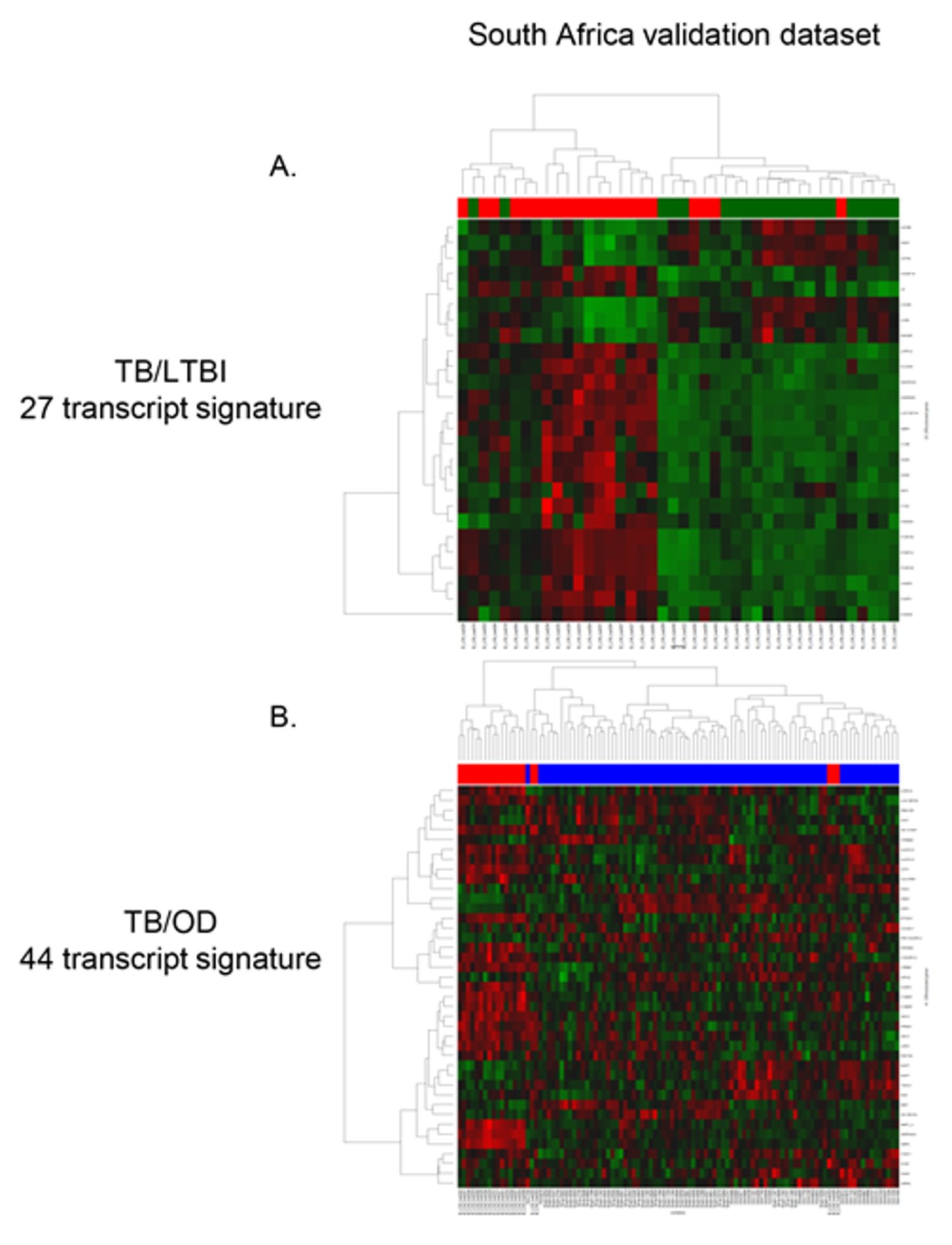

Supplement: Figure S4 — Heatmaps showing clustering of the independent South African validation dataset based on the TB/LTBI and TB/OD signatures. Clustering of TB versus LTBI based on the TB/LTBI 27 transcript signature (A) and TB/OD 44 transcript signature (B) applied to the independent South African validation datasets of Berry et al. [25]. Patients are represented as columns (red are patients with TB, green are LTBI, blue are OD) and individual transcripts are shown in rows (transcripts shown in red are up-regulated and those in green are down-regulated). (TIF) [file pmed.1001538.s004.tif]

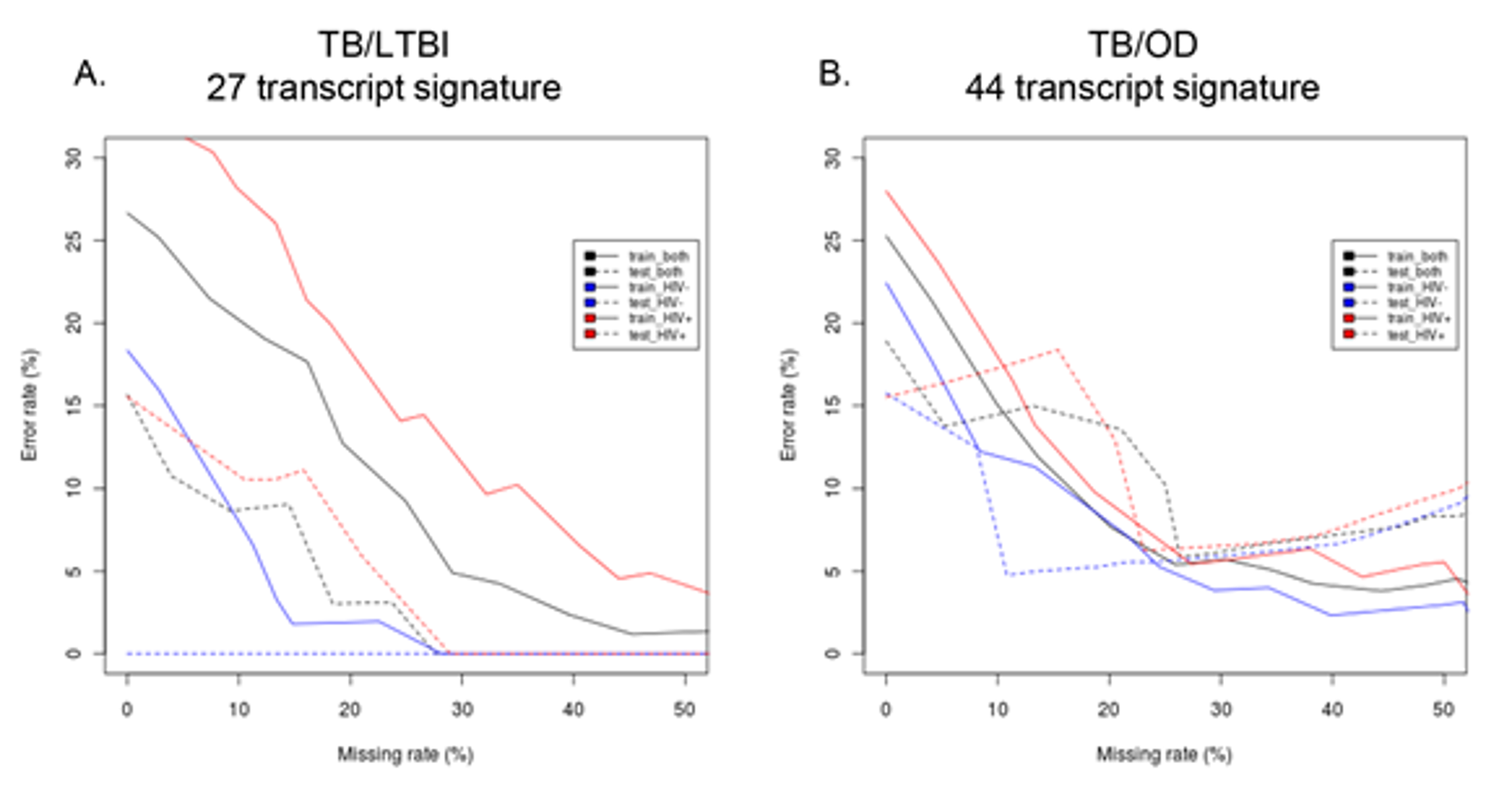

Supplement: Figure S5 — Calculating the error rate of the classifiers. The error rate of classification is presented in relation to the percentage of unclassified samples. We present the error rate of the classifier for the different groups using the 27 TB/LTBI and 44 TB/OD transcript signatures in relation to the missing rate we accept (HIV+ patients in red, HIV− in blue and both HIV+ & HIV− in black; solid lines show the error rate for the training cohorts while dotted lines show the error rate for the test cohorts). (TIF) [file pmed.1001538.s005.tif]
