# Supplementary material for: Detection of Tuberculosis in HIV-Infected and -Uninfected African Adults Using Whole Blood RNA Expression Signatures: A Case-Control Study
Source: PLoS Med. 2013 Oct 22;10(10):e1001538. doi: 10.1371/journal.pmed.1001538 (PMC3805485; doi:10.1371/journal.pmed.1001538)
Supplement: Table S1 — The 27 transcript signature for distinguishing TB from LTBI. (DOC) [file pmed.1001538.s006.doc]

## **Table S1:** **The 27 transcript signature for distinguishing TB from LTBI.** 27 transcript signature for distinguishing TB from latent TB infection, including Illumina array/probe ID and direction of regulation.

| **Array ID** | **Gene Symbol** | **Probe ID** | **Direction of regulation*** |
| --- | --- | --- | --- |
| 70730 | GAS6 | ILMN_1779558 | Up |
| 130181 | ANKRD22 | ILMN_1799848 | Up |
| 360132 | LHFPL2 | ILMN_1747744 | Up |
| 520086 | FCGR1A | ILMN_2176063 | Up |
| 1300139 | GNG7 | ILMN_1728107 | Down |
| 1340241 | C5 | ILMN_1746819 | Up |
| 1440341 | C1QC | ILMN_1785902 | Up |
| 1510026 | FLVCR2 | ILMN_2204876 | Up |
| 1780440 | CD79A | ILMN_1659227 | Down |
| 2630195 | VAMP5 | ILMN_1809467 | Up |
| 2650605 | C4ORF18 | ILMN_1672124 | Up |
| 2710709 | FCGR1B | ILMN_2261600 | Up |
| 2810373 | FAM20A | ILMN_1812091 | Up |
| 2970397 | ZNF296 | ILMN_1693242 | Down |
| 3520601 | MPO | ILMN_1705183 | Up |
| 3780047 | GBP6 | ILMN_1756953 | Up |
| 3890400 | CXCR5 | ILMN_2337928 | Down |
| 4280632 | GAS6 | ILMN_1784749 | Up |
| 5570039 | LOC728744 | ILMN_1654389 | Up |
| 5570398 | FCGR1C | ILMN_3247506 | Up |
| 5890470 | CCR6 | ILMN_1690907 | Down |
| 5910019 | C1QB | ILMN_1796409 | Up |
| 5910632 | SMARCD3 | ILMN_2309180 | Up |
| 6060468 | S100A8 | ILMN_1729801 | Up |
| 6450594 | CD79B | ILMN_1710017 | Down |
| 6560156 | DUSP3 | ILMN_1797522 | Up |
| 6620209 | FCGR1B | ILMN_2391051 | Up |

* in TB patients in relation to patients with latent TB infection.
