# Supplementary material for: Detection of Tuberculosis in HIV-Infected and -Uninfected African Adults Using Whole Blood RNA Expression Signatures: A Case-Control Study
Source: PLoS Med. 2013 Oct 22;10(10):e1001538. doi: 10.1371/journal.pmed.1001538 (PMC3805485; doi:10.1371/journal.pmed.1001538)
Supplement: Table S2 — The 44 transcript signature for distinguishing TB from other diseases. (DOC) [file pmed.1001538.s007.doc]

## **Table S2:** **The 44 transcript signature for distinguishing TB from other diseases.** 44 transcript signature for distinguishing TB from other diseases, including Illumina array/probe ID and direction of regulation.

| **Array ID** | **Gene Symbol** | **Probe ID** | **Direction of regulation*** |
| --- | --- | --- | --- |
| 130086 | CYB561 | ILMN_1771179 | Up |
| 150224 | LOC196752 | ILMN_1803743 | Up |
| 270039 | HM13 | ILMN_1766269 | Up |
| 360132 | LHFPL2 | ILMN_1747744 | Up |
| 380541 | PPPDE2 | ILMN_1737580 | Up |
| 450132 | RBM12B | ILMN_1805778 | Up |
| 450379 | PRDM1 | ILMN_2294784 | Up |
| 540041 | CASC1 | ILMN_1708983 | Up |
| 840446 | CYB561 | ILMN_2378376 | Up |
| 1030433 | CALML4 | ILMN_1815707 | Up |
| 1050360 | HLA-DPB1 | ILMN_1749070 | Up |
| 1070477 | ALDH1A1 | ILMN_2096372 | Up |
| 1110592 | EBF1 | ILMN_1778681 | Down |
| 1170332 | AAK1 | ILMN_1688755 | Up |
| 1580437 | PGA5 | ILMN_1717572 | Down |
| 1690184 | RNF19A | ILMN_1812327 | Up |
| 2000682 | HS.131087 | ILMN_1916292 | Down |
| 2030309 | SERPING1 | ILMN_1670305 | Up |
| 2260349 | MIR1974 | ILMN_3308961 | Up |
| 2340241 | IMPA2 | ILMN_2094061 | Down |
| 2350114 | GJA9 | ILMN_1710161 | Up |
| 2850315 | ORM1 | ILMN_1696584 | Down |
| 3120475 | MAP7 | ILMN_2216815 | Down |
| 3130600 | BTN3A1 | ILMN_1802708 | Up |
| 3310504 | PDK4 | ILMN_1684982 | Down |
| 3360553 | RP5-1022P6.2 | ILMN_1701111 | Down |
| 3780047 | GBP6 | ILMN_1756953 | Up |
| 3840053 | UGP2 | ILMN_1671969 | Up |
| 4070524 | CERKL | ILMN_1801091 | Up |
| 4290619 | CREB5 | ILMN_1728677 | Up |
| 4560047 | CD74 | ILMN_1761464 | Up |
| 4570164 | LOC389386 | ILMN_3215715 | Up |
| 4640768 | VPREB3 | ILMN_1700147 | Down |
| 4670458 | SEPT4 | ILMN_1776157 | Up |
| 5260161 | HS.162734 | ILMN_1893697 | Down |
| 5270753 | ARG1 | ILMN_1812281 | Down |
| 5290100 | MAK | ILMN_1803984 | Down |
| 5820491 | MAP7 | ILMN_1712719 | Down |
| 6380681 | C19ORF12 | ILMN_1664920 | Up |
| 6510754 | ALDH1A1 | ILMN_1709348 | Up |
| 6560156 | DUSP3 | ILMN_1797522 | Up |
| 6760056 | LOC100133800 | ILMN_3287952 | Up |
| 6760471 | TMCC1 | ILMN_1677963 | Down |
| 7210110 | HM13 | ILMN_2236655 | Up |

***** in TB patients in relation to patients with other diseases.
