# Supplementary material for: Detection of Tuberculosis in HIV-Infected and -Uninfected African Adults Using Whole Blood RNA Expression Signatures: A Case-Control Study
Source: PLoS Med. 2013 Oct 22;10(10):e1001538. doi: 10.1371/journal.pmed.1001538 (PMC3805485; doi:10.1371/journal.pmed.1001538)
Supplement: Table S3 — The 53 transcript signature for detecting TB from non-TB (i.e., LTBI and OD). (DOC) [file pmed.1001538.s008.doc]

## **Table S3: The 53 transcript signature for detecting TB from non-TB (i.e. LTBI and OD).** 53 transcript signature for distinguishing TB from other diseases, including Illumina array/probe ID and direction of regulation.

| **Array ID** | **Gene Symbol** | **Probe ID** | **Direction of regulation*** |
| --- | --- | --- | --- |
| 70730 | GAS6 | ILMN_1779558 | Up |
| 130086 | CYB561 | ILMN_1771179 | Up |
| 130181 | ANKRD22 | ILMN_1799848 | Up |
| 360132 | LHFPL2 | ILMN_1747744 | Up |
| 380541 | PPPDE2 | ILMN_1737580 | Up |
| 520086 | FCGR1A | ILMN_2176063 | Up |
| 540041 | CASC1 | ILMN_1708983 | Up |
| 840446 | CYB561 | ILMN_2378376 | Up |
| 870408 | IL15 | ILMN_1724181 | Up |
| 1030433 | CALML4 | ILMN_1815707 | Up |
| 1070477 | ALDH1A1 | ILMN_2096372 | Up |
| 1090497 | CREG1 | ILMN_1680624 | Up |
| 1110592 | EBF1 | ILMN_1778681 | Down |
| 1300139 | GNG7 | ILMN_1728107 | Down |
| 1510364 | GBP5 | ILMN_2114568 | Up |
| 1580437 | PGA5 | ILMN_1717572 | Down |
| 1660021 | RNU4ATAC | ILMN_3240594 | Up |
| 1940274 | IFI27L2 | ILMN_1740319 | Up |
| 2000682 | Hs.131087 | ILMN_1916292 | Down |
| 2340682 | UHMK1 | ILMN_2096012 | Up |
| 2680136 | SIGLEC11 | ILMN_1674593 | Up |
| 2970747 | DEFA3 | ILMN_2165289 | Up |
| 3130600 | BTN3A1 | ILMN_1802708 | Up |
| 3190113 | VPS13B | ILMN_2268409 | Up |
| 3420259 | MIR21 | ILMN_3310840 | Up |
| 3780047 | GBP6 | ILMN_1756953 | Up |
| 3840053 | UGP2 | ILMN_1671969 | Up |
| 3840753 | HEY1 | ILMN_1788203 | Down |
| 3890400 | CXCR5 | ILMN_2337928 | Down |
| 4290619 | CREB5 | ILMN_1728677 | Up |
| 4540239 | DEFA1 | ILMN_2193213 | Up |
| 4560047 | CD74 | ILMN_1761464 | Up |
| 4570164 | LOC389386 | ILMN_3215715 | Up |
| 4640768 | VPREB3 | ILMN_1700147 | Down |
| 4670113 | LOC90925 | ILMN_1794927 | Down |
| 4670458 | SEPT4 | ILMN_1776157 | Up |
| 4860128 | DEFA1B | ILMN_1725661 | Up |
| 5260161 | Hs.162734 | ILMN_1893697 | Down |
| 5570398 | FCGR1C | ILMN_3247506 | Up |
| 5720180 | FZD2 | ILMN_1653711 | Up |
| 5820491 | MAP7 | ILMN_1712719 | Down |
| 6330471 | BLK | ILMN_1668277 | Down |
| 6380040 | COL9A2 | ILMN_1685122 | Down |
| 6380338 | POLB | ILMN_1767894 | Up |
| 6400414 | LOC650546 | ILMN_1814812 | Up |
| 6510754 | ALDH1A1 | ILMN_1709348 | Up |
| 6560156 | DUSP3 | ILMN_1797522 | Up |
| 6590646 | FAM26F | ILMN_2066849 | Up |
| 6620161 | SPIB | ILMN_2143314 | Down |
| 6620209 | FCGR1B | ILMN_2391051 | Up |
| 6760471 | TMCC1 | ILMN_1677963 | Down |
| 6760593 | OSBPL10 | ILMN_1669497 | Down |
| 7150170 | DEFA1B | ILMN_2102721 | Up |

* in TB patients in relation to patients with latent TB infection and other diseases.
