# Supplementary material for: Detection of Tuberculosis in HIV-Infected and -Uninfected African Adults Using Whole Blood RNA Expression Signatures: A Case-Control Study
Source: PLoS Med. 2013 Oct 22;10(10):e1001538. doi: 10.1371/journal.pmed.1001538 (PMC3805485; doi:10.1371/journal.pmed.1001538)
Supplement: Table S4 — Number of patients per group and calls of DRS classification per group. (DOC) [file pmed.1001538.s009.doc]

## **Table S4: Number of patients per group and calls of DRS classification per group.** Values of sensitivity, specificity and their confidence intervals are presented in Table 3.

|  | **South Africa/Malawi test cohort** | | | **Validation dataset** |
| --- | --- | --- | --- | --- |
|  | **HIV+/- (95% CI)** | **HIV- (95% CI)** | **HIV+ (95% CI)** | **HIV- (95% CI)** |
| **TB vs. latent TB infection (27 TB/LTBI transcript signature)** |  |  |  |  |
| Number of patients | n_ALL_=76; n_TB_=37; n_LTBI_=39 | n_ALL_=38; n_TB_=19; n_LTBI_=19 | n_ALL_=38; n_TB_=18; n_LTBI_=20 | n_ALL_=51; n_TB_=20; n_LTBI_=31 |
| Positive calls by DRS / Positive by gold standard | [35/37] | [19/19] | [17/18] | [19/20] |
| Negative calls by DRS / Negative by gold standard | [35/39] | [19/19] | [18/20] | [29/31] |
| **TB vs. Other Diseases (44 TB/OD transcript signature)** |  |  |  |  |
| Number of patients | n_ALL_=76; n_TB_=42; n_OD_=34 | n_ALL_=37; n_TB_=22; n_OD_=15 | n_ALL_=39; n_TB_=20; n_OD_=19 | n_ALL_=102; n_TB_=20; n_OD_=83 |
| Positive calls by DRS / Positive by gold standard | [39/42] | [20/22] | [19/20] | [20/20] |
| Negative calls by DRS / Negative by gold standard | [30/34] | [14/15] | [16/19] | [80/83] |
