# Supplementary material for: Detection of Tuberculosis in HIV-Infected and -Uninfected African Adults Using Whole Blood RNA Expression Signatures: A Case-Control Study
Source: PLoS Med. 2013 Oct 22;10(10):e1001538. doi: 10.1371/journal.pmed.1001538 (PMC3805485; doi:10.1371/journal.pmed.1001538)
Supplement: Table S5 — Comparison of classification achieved using elastic net derived linear classifier and disease risk score for every pairwise comparison. (DOC) [file pmed.1001538.s010.doc]

## **Table S5: Comparison of classification achieved using elastic net derived linear classifier and disease risk score for every pairwise comparison.** Classification achieved using elastic net derived linear classifier and disease risk score with the 27 transcript set identified for TB versus latent TB infection, and the 44 transcript set for TB versus Other Diseases when applied to the HIV-uninfected (HIV-) and HIV-infected (HIV+) training and test cohorts.

|  | **Elastic net** | | | | | | **Disease risk score** | | | | | |
| --- | --- | --- | --- | --- | --- | --- | --- | --- | --- | --- | --- | --- |
|  | **HIV+/-** | | **HIV-** | | **HIV+** | | **HIV+/-** | | **HIV-** | | **HIV+** | |
|  | **Training** | **Test** | **Training** | **Test** | **Training** | **Test** | **Training** | **Test** | **Training** | **Test** | **Training** | **Test** |
|  | **(95% CI)** | **(95% CI)** | **(95% CI)** | **(95% CI)** | **(95% CI)** | **(95% CI)** | **(95% CI)** | **(95% CI)** | **(95% CI)** | **(95% CI)** | **(95% CI)** | **(95% CI)** |
| **TB vs. LTBI (27 TB/LTBI transcript signature)** |  |  |  |  |  |  |  |  |  |  |  |  |
| Area under the curve | 97% | 97% | 99% | 100% | 95% | 96% | 95% | 98% | 98% | 100% | 92% | 97% |
|  | (95-98) | (94-99) | (98-100) | (100-100) | (91-98) | (91-100) | (93-97) | (95-100) | (97-100) | (100-100) | (88-96) | (95-100) |
| Sensitivity | 87% | 89% | 84% | 84% | 89% | 94% | 87% | 95% | 91% | 100% | 81% | 94% |
|  | (81-92) | (78-97) | (75-92) | (68-100) | (81-96) | (83-100) | (81-92) | (87-100) | (85-97) | (100-100) | (72-90) | (83-100) |
| Specificity | 91% | 90% | 99% | 100% | 84% | 80% | 87% | 90% | 89% | 100% | 86% | 90% |
|  | (86-96) | (80-97) | (96-100) | (100-100) | (75-91) | (60-95) | (81-92) | (80-97) | (81-95) | (100-100) | (77-94) | (75-100) |
| **TB vs. Other Diseases (44 TB/OD transcript signature)** |  |  |  |  |  |  |  |  |  |  |  |  |
| Area under the curve | 97% | 94% | 97% | 95% | 97% | 94% | 96% | 95% | 97% | 96% | 95% | 94% |
|  | (95-98) | (88-99) | (94-100) | (88-100) | (94-99) | (84-100) | (94-98) | (89-99) | (94-99) | (89-100) | (92-98) | (83-100) |
| Sensitivity | 93% | 83% | 95% | 82% | 92% | 85% | 88% | 93% | 89% | 91% | 86% | 95% |
|  | (90-97) | (71-93) | (89-99) | (64-96) | (86-97) | (70-100) | (82-93) | (83-100) | (83-96) | (77-100) | (78-92) | (85-100) |
| Specificity | 89% | 97% | 90% | 100% | 88% | 95% | 87% | 88% | 88% | 93% | 86% | 84% |
|  | (83-94) | (91-100) | (82-96) | (100-100) | (80-95) | (84-100) | (82-92) | (74-97) | (79-96) | (80-100) | (78-95) | (68-100) |
