# Supplementary material for: Detection of Tuberculosis in HIV-Infected and -Uninfected African Adults Using Whole Blood RNA Expression Signatures: A Case-Control Study
Source: PLoS Med. 2013 Oct 22;10(10):e1001538. doi: 10.1371/journal.pmed.1001538 (PMC3805485; doi:10.1371/journal.pmed.1001538)
Supplement: Table S6 — Classification achieved using the disease risk score applied to the South African/Malawi HIV-uninfected (HIV−) and HIV-infected (HIV+) test cohort and validation dataset with confidence intervals calculated using the exact binomial method (Text S1). (DOC) [file pmed.1001538.s011.doc]

**Table S6:** **Classification achieved using the disease risk score applied to the South African/Malawi HIV-uninfected (HIV-) and HIV-infected (HIV+) test cohort and validation dataset with confidence intervals calculated using the exact binomial method (Text S1).**

|  | **South Africa/Malawi test cohort** | | | **Validation dataset** |
| --- | --- | --- | --- | --- |
|  | **HIV+/-** | **HIV-** | **HIV+** | **HIV-** |
|  | **(95% CI)** | **(95% CI)** | **(95% CI)** | **(95% CI)** |
| **TB vs. latent TB infection (27 TB/LTBI transcript signature)** | | | | |
| Sensitivity (mean) | 95% | 100% | 94% | 95% |
|  | (82-99) | (82-100) | (73-100) | (75-100) |
| Specificity (mean) | 90% | 100% | 90% | 94% |
|  | (76-96) | (82-100) | (68-99) | (79-99) |
| **TB vs. Other Diseases (44 TB/OD transcript signature)** | | | | |
| Sensitivity (mean) | 93% | 91% | 95% | 100% |
|  | (81-99) | (71-99) | (75-100) | (83-100) |
| Specificity (mean) | 88% | 93% | 84% | 96% |
|  | (73-97) | (68-100) | (60-97) | (90-99) |
