# Supplementary material for: Detection of Tuberculosis in HIV-Infected and -Uninfected African Adults Using Whole Blood RNA Expression Signatures: A Case-Control Study
Source: PLoS Med. 2013 Oct 22;10(10):e1001538. doi: 10.1371/journal.pmed.1001538 (PMC3805485; doi:10.1371/journal.pmed.1001538)
Supplement: Table S7 — Positive and negative predictive values for the classification achieved using the disease risk score applied to the South African/Malawi HIV-uninfected (HIV−) and HIV-infected (HIV+) test cohort and validation dataset. (DOC) [file pmed.1001538.s012.doc]

**Table S7:** **Positive and Negative predictive values for the classification achieved using the disease risk score applied to the South African/Malawi HIV-uninfected (HIV-) and HIV-infected (HIV+) test cohort and validation dataset.**

|  | **South Africa/Malawi test cohort** | | | | | | **Validation dataset** | |
| --- | --- | --- | --- | --- | --- | --- | --- | --- |
|  | **HIV+/-** | | **HIV-** | | **HIV+** | | **HIV-** | |
|  | **PPV (95% CI)** | **NPV (95% CI)** | **PPV (95% CI)** | **NPV (95% CI)** | **PPV (95% CI)** | **NPV (95% CI)** | **PPV (95% CI)** | **NPV (95% CI)** |
| **TB vs. LTBI (27 TB/LTBI transcript signature)** |  |  |  |  |  |  |  |  |
| 20% prevalence | 70% (50-89) | 99% (97-100) | 100% (100-100) | 100% (100-100) | 70% (42-99) | 98% (96-100) | 79% (56-100) | 99% (96-100) |
| 58% prevalence | 93% (86-99) | 92% (83-100) | 100% (100-100) | 100% (100-100) | 93% (84-100) | 92% (78-100) | 95% (89-100) | 93% (81-100) |
| **TB vs. Other Diseases (44 TB/OD transcript signature)** |  |  |  |  |  |  |  |  |
| 20% prevalence | 66% (46-87) | 98% (96-100) | 77% (44-100) | 98% (94-100) | 60% (35-85) | 99% (96-100) | 87% (75-100) | 100% (100-100) |
| 58% prevalence | 92% (84-99) | 90% (80-100) | 95% (86-100) | 88% (74-100) | 89% (79-99) | 92% (79-100) | 97% (95-100) | 100% (100-100) |
