# Supplementary material for: Detection of Tuberculosis in HIV-Infected and -Uninfected African Adults Using Whole Blood RNA Expression Signatures: A Case-Control Study
Source: PLoS Med. 2013 Oct 22;10(10):e1001538. doi: 10.1371/journal.pmed.1001538 (PMC3805485; doi:10.1371/journal.pmed.1001538)
Supplement: Table S8 — Performance of the smaller signatures when applied to the South Africa/Malawi test set. (DOC) [file pmed.1001538.s013.doc]

**Table S8: Performance of the smaller signatures when applied to the South Africa/Malawi test set.**

|  | **South Africa/Malawi test cohort** | |
| --- | --- | --- |
|  | Sensitivity (95% CI) | Specificity (95% CI) |
| **TB vs. latent TB infection** | | |
| 27 transcript signature | 95% (87-100) | 90% (80-97) |
| 21 transcript signature | 89% (78-97) | 89% (79-97) |
| **TB vs. other diseases** | | |
| 44 transcript signature | 93% (83-100) | 88% (74-97) |
| 29 transcript signature | 83% (69-93) | 88% (75-97) |
