# Supplementary material for: Detection of Tuberculosis in HIV-Infected and -Uninfected African Adults Using Whole Blood RNA Expression Signatures: A Case-Control Study
Source: PLoS Med. 2013 Oct 22;10(10):e1001538. doi: 10.1371/journal.pmed.1001538 (PMC3805485; doi:10.1371/journal.pmed.1001538)
Supplement: Table S9 — Classification achieved using the disease risk score applied to the South African/Malawi smear-negative patients with TB and the controls from the test cohort with confidence intervals calculated using the bootstrapping and the exact binomial method. (DOC) [file pmed.1001538.s014.doc]

**Table S9: Classification achieved using the disease risk score applied to the South African/Malawi smear-negative patients with TB and the controls from the test cohort with confidence intervals calculated using the bootstrapping and the exact binomial method.**

|  | **South Africa/Malawi smear negative TB**  **and controls from the test cohort** | |
| --- | --- | --- |
|  | Sensitivity (95% CI) | Specificity (95% CI) |
| **TB vs. latent TB infection**  n_ALL_=70; n_TB smear neg_=31; n_LTBI_=39 | | |
| Calls by DRS | [21/31] | [35/39] |
| Bootstrapping | 68% (52-84) | 90% (80-97) |
| Exact Binomial | 68% (49-83) | 90% (76-97) |
| **TB vs. other diseases**  n_ALL_=65; n_TB smear neg_=31; n_OD_=34 | | |
| Calls by DRS | [28/31] | [30/34] |
| Bootstrapping | 90% (81-100) | 88% (74-97) |
| Exact Binomial | 90% (74-98) | 88% (73-97) |
