# Supplementary material for: Detection of Tuberculosis in HIV-Infected and -Uninfected African Adults Using Whole Blood RNA Expression Signatures: A Case-Control Study
Source: PLoS Med. 2013 Oct 22;10(10):e1001538. doi: 10.1371/journal.pmed.1001538 (PMC3805485; doi:10.1371/journal.pmed.1001538)
Supplement: Text S1 — Appendix. (DOC) [file pmed.1001538.s015.doc]

**Text S1**

**Laboratory Methods**

*RNA sample extraction and processing*

Whole blood (2.5ml) was collected into PAXgene blood RNA tubes (PreAnalytiX), incubated for 2 hours, frozen at -20^o^C within 3 hours of collection, and then stored at -80^o^C. RNA was extracted using PAXgene blood RNA kits (PreAnalytiX) according to the manufacturer’s instructions at one site (Cape Town) to minimize any sample handling bias. The integrity and yield of the total RNA was assessed using an Agilent 2100 Bioanalyser and a NanoDrop 1000 spectrophotometer respectively. Total RNA was then shipped to the Genome Institute of Singapore. After quantification and quality control, biotin-labeled cRNA was prepared using Illumina TotalPrep RNA Amplification kits (Applied Biosystems) from 500ng RNA. Labeled cRNA was hybridized overnight to Human HT-12 V4 Expression BeadChip arrays (Illumina). After washing, blocking and staining, the arrays were scanned using an Illumina BeadArray Reader according to the manufacturer’s instructions. Using Genome Studio software the microarray images were inspected for artifacts and QC parameters were assessed. No arrays were excluded at this stage.

**Statistical Methods**

*Microarray analysis*

Mean raw intensity values for each probe were corrected for local background intensities and a robust spline normalisation (combining quantile normalisation and spline interpolation) was applied to each array. Expression values were transformed to a logarithmic scale (base 2), and for each transcript. Differential expression between patient groups was identified by fitting a linear model to each transcript using LIMMA . P-values were adjusted using the method of Benjamini and Hochberg . Transcripts with |log_2_ FC| >0.5 were taken forward to variable selection with elastic net . This threshold was chosen in order to ensure that differential expression for selected variables could be distinguished using the resolution of qtPCR. The α and λ parameters of elastic net, which control the size of the selected model, were optimized via ten-fold cross-validation (CV). The weights assigned by elastic net to the trained model were used within a linear regression model to classify samples in the test set.

*Disease risk score*

For each individual, we calculated the disease risk score using the minimal transcript selected sets for TB versus LTBI and TB versus OD. The score is based on subtracting the summed intensities of the down-regulated transcripts from the summed intensities of the up-regulated transcripts. The risk score was calculated on normalised intensities. The disease risk score for individual *i* is:


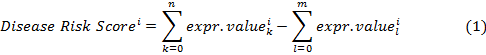


where: *n* the number of up-regulated number of transcripts in the signature in disease of interest compared to comparator group(s).

*m* the number of down-regulated number of transcripts in the signature in disease of interest compared to comparator group(s).

The threshold for the classification was calculated as the weighted average of risk score within each class (group of patients), with weights given as inverse of the standard deviation of the score within each class. The threshold for the classification between group *u* and *v* is shown below:


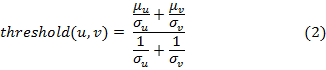


where: *μ* average of the disease risk score in the group.

*σ* standard deviation of the disease risk score in the group.

We further explored the effect of adjusting the threshold for the disease risk score in assigning individual patients to TB or non-TB groups. By accepting a percentage of patients as “not classified” by our test and thus requiring further investigation, the majority of patients under investigation are accurately assigned, suggesting that the score might be used as an initial screening “rule in/rule out” approach to TB diagnosis. To calculate the indeterminate zone, we calculated the lower and upper threshold which were calculated as the weighted average with weights given by
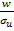
,
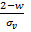
respectively:


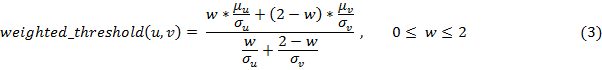


When w=1 the formula is equivalent to the main threshold formula.

Further evaluation of how a test based on our signatures might be used operationally and assessment of its cost effectiveness is needed if our TB versus LTBI and TB versus OD signatures are to be translated into simple cheap assays.

*Evaluation of the classification of the disease risk score (DRS) and the signatures*

To evaluate the performance of the DRS as a classifier we used different measures (AUC, sensitivity, specificity, PPV, NPV, and likelihood ratios).

The calculation of the confidence intervals for the area under a receiver operating characteristic curve (AUC), the sensitivity and the specificity was based on a non-parametric stratified bootstrap resampling (each replicate contained the same number of cases and controls as the original sample) , with 2000 bootstraps, as recommended by Carpenter et al. . We also employed the exact binomial to calculate the confidence intervals (Table S6).

We used the estimated sensitivity and specificity to calculate the positive and negative predictive values (PPV and NPV) using the following formulas:


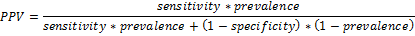


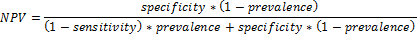


and interpreting the prevalence as “the probability before the test is carried out that the subject has the disease” as suggested by D. Altman . In this case, we assumed a clinical setting, such as the one used to recruit samples in Malawi, in which approximately 58% of patients with suspected TB had culture confirmed TB (254 TB confirmed cases / 437 patients with suspected TB), as well as calculating more conservative values assuming a prevalence of 20% (as a more typical proportion would be 15%-25% in quality controlled laboratories in primary care settings in high-burden countries in sub-Saharan Africa). PPV and NPV can be interpreted as the probability that a sample with a positive test has active TB, and the probability that a sample with a negative test result does not have active TB respectively, and as such represent the diagnostic value of a test (Table S7). We also report positive and negative likelihood ratios along with their confidence intervals employing the method described in (Table 3).

*Smaller sets of transcripts*

Although the models suggested by elastic net were the smallest ones to provide us with the best classification, we wanted to further explore the performance of even smaller lists of transcripts. Instead of optimizing via ten-fold cross-validation (CV) both the α and λ parameters of elastic net which control the size of the selected model, we used α=1 which is the penalty for lasso that gives smaller models. Then, within the cross-validation step of choosing λ, we forced the penalty to be such that the error would remain within one standard deviation of minimum error. This process resulted in 21 transcripts for the TB versus LTBI comparison (12 overlapping with the 27 transcript signature) and 29 transcripts for the TB versus OD comparison (14 overlapping with the 44 transcript signature). Smaller models have reduced sensitivity (6% -10% lower than the original models) while specificity remained the same (Table S8). When the DRS was calculated, sensitivity and specificity were 89%, 95% CI (78-97) and 89%, 95% CI (79-97) respectively for the TB versus LTBI comparison. As for the TB versus OD comparison, when the DRS was calculated, sensitivity and specificity were 83%, 95% CI (69-93) and 88%, 95% CI (75-97) respectively. Smaller models have mainly reduced sensitivity.

*Smear-negatives*

We have included 31 smear-negative patients with TB (with definite negative smear status) in the analysis of the adult cohort (7 TB HIV-uninfected and 24 TB HIV-infected). The TB/LTBI and the TB/OD DRSs were applied to these patients and as controls we used the LTBI and OD patients from the test set, while maintaining the same threshold. The performance of the TB/LTBI signature was comparable to the performance in the HIV-infected group and the performance of the TB/OD signature was almost the same as in the larger smear-negative and smear-positive group. Confidence intervals for the sensitivity and specificity of smear-negative patients with TB were calculated using both the bootstrapping and the exact binomial method (Table S9). These confidence intervals overlapped the corresponding CIs for the larger smear-positive and smear-negative group.

*Analysis of validation datasets*

For validation of the performance of the disease risk score based on the TB/LTBI 27 transcript signature and TB/OD 44 transcript signature, we used the whole blood expression dataset of Berry et al. generated using Illumina HT12 V3 Beadarrays comparing TB with LTBI and other infections in an Africa cohort (accession series GSE19491). The cohort comprised HIV-uninfected individuals; no TB HIV-infected adult cohort was publically available. For each testing dataset (SA GSE19442, OD GSE22098), both quantile and robust spline normalisation were applied separately to the arrays and the data was log transformed – however the results were the same regardless of normalisation method.

For the evaluation of the performance of our TB/LTBI 27 transcript signature, we used TB and LTBI patients in the SA testing set (TB n=20, LTBI n=31). The probe ILMN_3247506 (FCGR1C) was not on the HT12 V3 beadarray. For the evaluation of the performance of our 44 TB/OD transcript signature, we used TB patients from the SA testing set (TB n=20) and OD patients that did not include patients with systemic lupus erythematosus as they were judged to be a rare disease in an African setting (n=82). The probes ILMN_3287952 (LOC100133800), ILMN_3215715 (LOC389386) and ILMN_3308961 (MIR1974) were not on the HT12 V3 Beadchip.

For testing the performance of the reported 393 TB versus LTBI signature and the 86 TB versus OD signature on our African dataset, the disease risk score was calculated with these signatures as previously described, although 7 probes in the reported signatures were not present on the HT-12 V4 Beadchip (TB vs. LTBI 6 probes, TB vs. OD 1 probe).

In order to compare directly the differences of the performance of our signatures to the signatures presented in the Berry et al., we calculated the differences of the means of the measures of classification (namely the AUC, the sensitivity and the specificity) on our test set along with their 95% confidence intervals, using the following mathematical formulas:


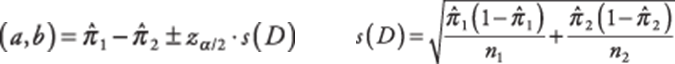


**References**

1. Schmid R, Baum P, Ittrich C, Fundel-Clemens K, Huber W, et al. (2010) Comparison of normalization methods for Illumina BeadChip HumanHT-12 v3. BMC Genomics 11: 349.

2. Smyth GK (2004) Linear models and empirical bayes methods for assessing differential expression in microarray experiments. Stat Appl Genet Mol Biol 3(1): Article 3.

3. Benjamini Y, Hochberg Y (1995) Controlling the False Discovery Rate - a Practical and Powerful Approach to Multiple Testing. Journal of the Royal Statistical Society Series B-Methodological 57: 289-300.

4. Zou H, Hastie T (2005) Regularization and variable selection via the elastic net. Journal of the Royal Statistical Society Series B-Statistical Methodology 67: 301-320.

5. Robin X, Turck N, Hainard A, Tiberti N, Lisacek F, et al. (2011) pROC: an open-source package for R and S+ to analyze and compare ROC curves. BMC Bioinformatics 12: 77.

6. Carpenter J, Bithell J (2000) Bootstrap confidence intervals: when, which, what? A practical guide for medical statisticians. Stat Med 19: 1141-1164.

7. Clopper CJ, Pearson ES (1934) The use of confidence or fiducial limits illustrated in the case of the binomial. Biometrika 26: 404-413.

8. Altman DG, Bland JM (1994) Diagnostic tests 2: Predictive values. BMJ 309: 102.

9. Simel DL, Samsa GP, Matchar DB (1991) Likelihood ratios with confidence: sample size estimation for diagnostic test studies. J Clin Epidemiol 44: 763-770.

10. Berry MP, Graham CM, McNab FW, Xu Z, Bloch SA, et al. (2010) An interferon-inducible neutrophil-driven blood transcriptional signature in human tuberculosis. Nature 466: 973-977.

# ILULU Consortium members

**Institute of Infectious Diseases and Molecular Medicine, University of Cape Town** Nonzwakazi Bangani, Lizl Bashe, Melina Carr, Hannah P. Gideon, Rene Goliath, Yekiwe Hlombe, Vanessa January, Bekekile Kwaza, Suzaan Marais, Marc Mendelson, Tolu Oni, Fadheela Patel, Ronnett Seldon, Relebohile Tsekela, Katalin A. Wilkinson, Robert J. Wilkinson, Kathryn Wood; **London School of Hygiene and Tropical Medicine/Karonga Prevention Study** Lyn Ambrose, Amelia C. Crampin, Hazel M. Dockrell, Neil French, Lumbani Munthali, Bagrey Ngwira, Amos Phiri, Femia Zgambo; **Red Cross War Memorial Children’s Hospital, University of Cape Town** Margaret Cooper, Brian Eley, Mabel Gcuwa, Spasina King, Glynis Kossew, Karen McCabe, Wonita Petersen, Sandra Pienaar, Vashini Pillay; **Liverpool School of Tropical Medicine/Malawi-Liverpool-Wellcome Trust Clinical Research Programme**, **University of Malawi College of Medicine** Benjamin Allubha, George Chagaluka, Angeziwa Chunga, Janet Dube, Robert S. Heyderman, Annie Joabe, Martha Kalemba, Anne Kerr, Monica Matola, Rachel Mlotha, Agnes Mwale, David Mzinza; **Brighton and Sussex Medical School, University of Sussex** Suzanne T. Anderson, Gillian Baker, Claire M. Banwell, Terry Bishop, Natalie Chaplin, Julian Golland, Florian Kern, Susan Poore, Jayne Wellington; **Imperial College London** Andrew J. Brent, Lachlan J. Coin, Hariklia Eleftherohorinou, Shea Hamilton, Myrsini Kaforou, Paul R. Langford, Michael Levin, Stephanie Menikou, Victoria J. Wright.
